# Age and Gender-related Variations of Molecular and Phenotypic Parameters in A Cohort of Sicilian Population: from Young to Centenarians

**DOI:** 10.14336/AD.2021.0226

**Published:** 2021-10-01

**Authors:** Anna Aiello, Giulia Accardi, Stefano Aprile, Rosalia Caldarella, Ciriaco Carru, Marcello Ciaccio, Immaculata De Vivo, Caterina Maria Gambino, Mattia Emanuela Ligotti, Sonya Vasto, Angelo Zinellu, Calogero Caruso, Filippa Bono, Giuseppina Candore

**Affiliations:** ^1^Laboratory of Immunopathology and Immunosenescence, Department of Biomedicine, Neuroscience and Advanced Diagnostic, University of Palermo, Palermo, Italy.; ^2^Unit of Transfusion Medicine, San Giovanni di Dio Hospital, Agrigento, Italy.; ^3^Department of Laboratory Medicine, “P. Giaccone” University Hospital, Palermo, Italy.; ^4^Department of Biomedical Sciences, University of Sassari, Sassari, Italy.; ^5^Unit of Clinical Biochemistry, Clinical Molecular Medicine, and Laboratory Medicine, Department of Biomedicine, Neuroscience and Advanced Diagnostic, University of Palermo, Palermo, Italy.; ^6^Department of Epidemiology, Harvard T.H. Chan School of Public Health, Boston, MA, USA.; ^7^Department of Biological, Chemical and Pharmaceutical Sciences and Technologies, University of Palermo, Palermo, Italy.; ^8^Department of Economics, Business and Statistics, University of Palermo, Palermo, Italy.

**Keywords:** aging, centenarian, gender, inflammation, longevity, phenotype

## Abstract

People are living longer, but lifespan increase does not coincide with a boost in health-span. Thus, improving the quality of life of older people is a priority. Centenarians reach extreme longevity in a relatively good health status, escaping or delaying fatal or strongly invalidating diseases. Therefore, studying processes involved in longevity is important to explain the biological mechanisms of health and well-being, since knowledge born from this approach can provide valuable information on how to slow aging. We performed the present study in a well characterized very homogeneous sample of 173 people from Western Sicily, to update existing literature on some phenotypic aspects of aging and longevity and to propose a range of values for older people. We classified 5 age groups, from young adults to centenarians, to understand the age and gender-related variations of the different parameters under study. We collected anamnestic data and performed anthropometric, bioimpedance, molecular, haematological, oxidative, and hematochemical tests, adopting a multidimensional analysis approach. An important evidence of the present study is that there are differences related to both age and gender in several biomarkers. Indeed, gender differences seem to be still poorly considered and inadequately investigated in aging as well as in other medical studies. Moreover, we often observed comparable parameters between young and centenarians rather than non-agenarians and centenarians, hypothesizing a sort of slowdown, almost followed by a reversal trend, in the decay of systemic deterioration. The study of centenarians provides important indications on how to slow aging, with benefits for those who are more vulnerable to disease and disability. The identification of the factors that predispose to a long and healthy life is of enormous interest for translational medicine in an aging world.

People worldwide are living longer. Nonagenarians and centenarians, *i.e*., long living individuals (LLIs), are among the most rapidly growing segments of the population (www.who.int/ageing/publications/global_health.pdf) [1]. According to United Nations estimates, in 2015 in the world there were nearly half a million centenarians, more than four times as many as in 1990 (https://population.un.org/wpp/). Moreover, this growth is expected to accelerate. Projections suggest that there will be 3.7 million centenarians across in 2050 (www.pewresearch.org/fact-tank/2016/04/21/worlds-centenarian-population-projected-to-grow-eightfold-by-2050/).

However, the increase in lifespan does not coincide with increase in health-span, *i.e*., the period of advanced life free from serious chronic diseases and disability [2].

Improving the quality of life of older people and slowing aging is becoming a priority due to the continuous increase in the number of this population [3]. This makes the studies of the processes involved in longevity of great importance. Beyond the study of age-related diseases, it is possible to follow another approach, called positive biology, investigating the causes of positive phenotypes to explain the biological mechanisms of health and well-being. The best example of a positive phenotype is represented by centenarians, *i.e*., people living 100 years or more, who reach extreme longevity in a relatively good health status, escaping fatal or strongly invalidating diseases lifelong [4]. The knowledge born from this approach could allow us to modulate the aging rate by providing valuable information on how to slow aging.

Naturally, it is not unexpected that centenarians might be frail, and that definition of their health status is methodologically difficult [2]. Reaching the age of 100, per se, does not necessarily indicate successful aging [5].

At this regard, three distinct morbidity profiles have been described, exploring the timing of most common age-related diseases in a sample of centenarians: the “escapers’’, who do not succumb to any age-related diseases; the “delayers’’, who postpone the onset of age-related diseases; and the “survivors’’, who outlived with disease [6].

Recently, in Southern Europe, it has shown that 27.1% of the centenarian population could be labelled as “survivors”, 26.5% as “delayers”, and 46.4% as “escapers” [7]. On the other hand, centenarians appear to outlive the risks for many of the conditions that are common causes of death, such as cancer and myocardial infarction, slowing aging [8].

Thus, new studies to define some phenotypic aspects (*i.e*., physical and functional) of very old people and centenarians are needed. Whereas the genetics of longevity is largely analysed via multinational research programmes, few observations are known about peculiar phenotypic aspects of centenarians, *i.e*., anthropometric features or specific range for hematochemical and oxidative stress values [9].

Thus, we performed a study in a well characterized very homogeneous sample of 173 people from Western Sicily, an Italian island in the centre of Mediterranean Sea, to update existing literature on some molecular and phenotypic aspects of aging and longevity and to propose a range of values for older people. We classified 5 age groups, *i.e*., young adults, adults, older adults, nonagenarians, and centenarians, in order to understand the age and gender-related variations of the different parameters under study. We grouped the last two classes as LLIs. We collected anamnestic data and performed anthropometric, bioimpedance, molecular, haematological, oxidative, and Hematochemical tests, adopting a multidimensional analysis approach.

## MATERIALS AND METHODS

### Study design, participants and anamnestic data

A total of 173 subjects of European ancestry were enrolled in Western Sicily within the project “Discovery of molecular and genetic/epigenetic signatures underlying resistance to age-related diseases and comorbidities (DESIGN, 20157ATSLF)”, funded by the Italian Ministry of Education, University and Research, from June 2017 to March 2020. The population was divided in five age groups, *i.e*., young adults (29 subjects, age range 18-39 years old), adults (40 subjects, age range 40-64 years old), older adults (54 subjects, age range 65-89 years old), nonagenarians (27 subjects, age range 90-99 years old), and centenarians (23 subjects, age range 100-111 years old). We selected healthy people, considering the age physiological deterioration of organs and systems, including deafness, visual problems, and if they have no more than one invalidating condition. Moreover, we included cognitive-performant individuals only (although not completely). Thus, we excluded people with chronic invalidating diseases, such as neoplastic and autoimmune ones, as well as with acute disease, such as infectious, and with severe dementia. The majority of recruited subjects signed an informed consent before the enrolment. For three subjects, the caregiver provided for it, due to the visual problems of the participants.

To respect privacy, everyone was identified with an alphanumeric code and the data were managed using a database accessible exclusively by researchers involved in the project. The study protocol, conducted in accordance with the Declaration of Helsinki and its amendments, was approved by the Ethic Committee of Palermo University Hospital (Nutrition and Longevity, No. 032017).

The enrolment was conducted at University of Palermo for young adults, adults, and older adults using social networks and word of mouth, whereas was conducted at home for nonagenarians and centenarians. This last procedure was chosen because of the difficulties for LLIs to go to our ambulatory. Most of our population of LLIs lived in small villages (most of them in the Madonie Mountains) with their family, so not in a big city, and in any case, they cannot drive and need a caregiver. For the recruitment of this group, we proceeded asking municipalities or general practitioners for the list of LLIs. Thereafter, contacting the family by a phone call to verify the consensus at participating in the study and the presence or absence of the inclusion and exclusion criteria, respectively. This kind of recruitment presents several limitations linked to the time and budget expenditure related to the home visit, often in the countryside that is difficult to reach from the town. But the advantage of home visit is to avoid any possible stress for LLIs and the possibility to study the context of living.

A team composed by demographers, biologists and physicians from University of Palermo administered to the participants a detailed questionnaire to collect demographic, clinical, and anamnestic data of interest as well as functional and cognitive information (see Supplementary Material). The questionnaire includes nine sections: main pathologies, drugs and smoking, cognitive status such as mini-mental state examination (MMSE), geriatric depression scale (GDS), activities of daily living (ADL), instrumental activity of daily living (IADL), sleep, and eating habits. The maximum MMSE score is 30 points. A score under 18 suggests severe dementia, 18 to 25 suggests moderate to mild dementia, 26 to 30 indicate normal cognitive ability. About GDS, we used the 15-items version, so the short one. The scores suggest respectively, 3±2 no depression, 7±3 mild depression, 12±2 severe depression. The MMSE, GDS, IADL, and ADL questionnaire sections were administered to LLIs only (for the Questionnaire see Supplementary material) [10,11] (www.oxfordmedicaleducation.com/geriatrics/mini-mental-state-examination-mmse/; www.apa.org/pi/about/publications/caregivers/practice-settings/assessment/tools/geriatric-depression).

Concerning eating habits, we collected data by an already used food frequency questionnaire [12,13].

Overnight fasting blood samples were obtained in the morning. The blood was collected in specific tubes containing EDTA or without additives to separate plasma, serum, and blood cells. Hematochemical and haematological tests were performed immediately. Serum and plasma were obtained after centrifugation and genomic DNA was extracted from leukocytes with a commercial kit. These samples were rapidly frozen and stored at -80°C for further analyses.

### Anthropometric and bioimpedance parameters

Anthropometric measures, including body weight and height, were performed with light dresses and barefoot and used to calculate the body mass index (BMI). The bioelectrical impedance analysis (BIA), that uses a single-frequency approach, was performed in the supine position. For the tetrapolar measurement, four skin electrodes were applied, one pair on the back of the hand and the other pair on the back of the ipsilateral foot. On the hand, there were placed one on the metacarpophalangeal joint of the third finger (injector electrode) and the other on the radio-ulnar joint (sensor electrode). On the foot, they were placed one on the metatarsophalangeal joint of the third toe (injector electrode) and the other on the tibio-tarsal joint (sensor electrode). The BIA device used was a portable Akern BIA 101 more suitable for at home visit, applying an alternating electrical current of 50 kHz (800 µA), not dangerous for tissues. The resistance, which reflects the volume of the water compartment, and the reactance, which is proportional to the cell mass in the body, both (in Ohm), and the phase angle (PhA, which indicates the relationship between resistance and reactance) were reported. PhA is a linear method of measuring the relationship between electric resistance and reactance and is an indicator of cell membrane functionality and of water compartment volume. The arc tangent value of the ratio of reactance versus electric resistance provides PhA values by BodygramPlus 1.1.4.4 software. The working principles, applications, merits, and demerits of BIA have been discussed in detail in Foster and Lukaski as well as in Bera [14,15].

### Molecular tests

Relative telomere length (RTL) was determined at the Dana Farber/Harvard Cancer Center Genotyping & Genetics for Population Sciences Facility (Boston, MA, USA), using a modified, high-throughput version of the real-time quantitative (Rtq) PCR-based telomere assay that was run on the Applied Biosystems 7900HT Sequence Detection System (Foster City, CA). 15 ng of genomic DNA was required for the protocol. The average RTL was determined as the copy-number ratio between telomere repeats and a single-copy (36B4) reference gene (T/S Ratio, -ΔCt). Leukocyte RTL is reported as the exponentiated T/S ratio corrected for a reference sample.

Although this assay measures RTL, the T/S ratio highly correlates with absolute TL provided by Southern Blot (r = 0.68-0.85; p<0.001) [16,17].

The single-nucleotide polymorphism (SNP) rs2802292 G-allele (G>T) of Forkhead box O3A (FOXO3A) gene was genotyped, using an amplification-refractory mutation system-polymerase chain reaction (ARMS-PCR), adopting registered and validated primers (certified at World International Property Organization, on 18/02/2010, n.WO 2010/019519 A2). Three genotypes were analysed: GG, GT, and TT. The size separation was conducted using agarose gel electrophoresis (2%).

**Table 1:** Anamnestic and bioimpedance parameters.

Variable (unit of measurement)	(a) Young adults (18-39 y.o.) N=29, M=13, W=16	(b) Adults (40-64 y.o.) N=40, M=21, W=19	(c) Older adults (65-89 y.o.) N=54, M=28, W=26	(d) Nonagenarians (90-99 y.o.) N=27, M=9, W=18	(e) Centenarians (100-111 y.o.) N=23, M=6, W=17	Age	Interaction		Gender
							M	W		
Blood pressure									
Systolic blood pressure (mmHg)	1 10.00 (70.00-140.00) N=29, (108.28±14.96) M=13, (113.85±16.85) W=16, (103.75±11.90)	1 30.00 (100.00-160.00) N=40, (128.50±13.27) M=21, (129.29±12.07) W=19, (127.63±14.75)	1 40.00 (100.00-160.00) N=54, (135.52±15.06) M=28, (134.03±13.98) W=26, (137.11±16.26)	140.00 (100.00-175.00) N=27, (141.15±17.80) M=9, (135.00±23.05) W=18, (140.56±17.90)	130.00 (50.00-180.00) N=23, (129.35±29.01) M=6, (133.33 ±23.59) W=17, (127.94 ±31.23)	(a vs b, c,d,e)	ns	ns	ns
Diastolic blood pressure (mmHg)	70.00 (55.00-130.00) N=29, (71.38±16.74) M=13, (74.61±15.74) W=16, (68.78±17.56)	80.00 (55.00-105.00) N=40, (79.00±9.75) M=21, (80.48±8.65) W=19, (77.27±10.85)	75.00 (60.00-100.00) N=54, (75.63±9.56) M=28, (75.14±10.39) W=26, (76.15±8.75)	70.00 (50.00-100.00) N=27, (79.74±11.41) M=9, (71.67±11.99) W=18, (70.28±8.75)	65.00 (50.00-145.00) N=23, (69.56 ±20.66) M=6, (67.50 ±10.84) W=17, (70.29 ±23.41)	ns	ns	ns	ns
Sleeping habits									
Mean hours/night	7.00 (6.00-9.00) N=26, (7.10±0.87) M=11, (6.96±0.93) W=15, (7.20±0.84)	6.50 (5.00-8.00) N=29, (6.60±0.92) M=16, (6.56±1.08) W=13, (6.65±0.72)	6.00 (2.50-10.00) N=36, (6.29±1.47) M=19, (6.42±1.62) W=17, (6.15±1.32)	7.00 (4.00-12.00) N=23, (7.46±2.43) M=9, (7.17±1.80) W=14, (7.64±2.80)	7.25 (4.00-12.00) N=16, (7.69 ±2.80) M=2, (7.25 ±3.89) W=14, (7.75 ±2.80)	ns	ns	ns	ns
Anthropometric feature									
BMI (Kg/m^2^)	22.50 (17.30-30.20) N=29, (22.68±3.19) M=13, (24.90±2.24) W=16, (20.88±2.71)	26.90 (21.30-40.70) N=39, (27.41±4.42) M=20, (27.22±2.97) W=19, (27.61±5.64)	27.90 (18.70-39.70) N=51, (28.23±4.63) M=26, (27.67±3.53) W=25, (28.80±5.56)	26.10 (18.20-40.90) N=22, (26.54±6.24) M=8, (27.27±6.36) W=14, (26.12±6.37)	22.65 (18.40-31.60) N=22, (23.45 ±3.68) M=6, (25.76 ±3.25) W=16, (22.58 ±3.53)	(a vs b,c,d) (c vs e)	ns	ns	ns
Bioimpedance features									
Rz (Ω)	620.00 (423.00-793.00) N=29, (613.75±93.31) M=13, (534.92±63.26) W=16, (677.81±57.36)	556.00 (376.00-774.00) N=39, (558.43±85.84) M=20, (505.65±56.35) W=19, (614.00±76.49)	560.00 (437.00-812.00) N=54, (561.05±67.49) M=28, (537.14±56.43) W=26, (586.80±69.91)	589.00 (438.00-812.00) N=22, (606.36±101.75) M=8, (564.62±113.60) W=14, (630.2±89.94)	612.00 (437.00-748.00) N=22, (614.36 ±75.58) M=6, (570.00 ±99.18) W=16, (631.00 ±60.30)	ns	ns	(a vs c)	=0.01
Xc (Ω)	63.00 (44.00-77.00) N=29, (61.68±9.07) M=13, (59.07±9.33) W=16, (63.81±8.55)	55.00 (36.00-69.00) N=39, (5.33±8.12) M=20, (53.10±8.11) W=19, (53.57±8.35)	47.00 (34.00-65.00) N=54, (47.57±7.04) M=28, (45.42±6.02) W=26, (49.88±7.44)	38.50 (28.00-75.00) N=22, (41.63±11.05) M=8, (40.12±8.18) W=14, (42.50±12.61)	32.00 (20.00-47.00) N=21, (31.85 ±7.15) M=6, (30.00 ±5.06) W=15, (32.60 ±7.87)	(a vs b,c,d,e) (b vs d,e) (c vs e)	ns	ns	=0.01
PhA (°)	5.80 (4.20-7.10) N=29, (5.79±0.72) M=13, (6.31±0.61) W=16, (5.38±0.51)	5.40 (3.70-6.90) N=39, (5.50±0.79) M=20, (6.01±0.64) W=19, (4.98±0.58)	4.80 (2.90-6.20) N=51, (4.84±0.71) M=26, (4.92±0.76) W=25, (4.78±0.69)	4.05 (2.40-6.70) N=22, (3.95±0.94) M=8, (4.10±0.61) W=14, (3.87±1.11)	2.90 (2.00-9.00) N=21, (3.24 ±1.43) M=6, (3.05 ±0.52) W=16, (3.33 ±1.63)	(a vs c,d,e) (b vs c,d,e)	(a vs c,d,e) (b vs c,d,e) (c vs e)	(a vs d,e) (b vs d,e) (c vs e)	=0.01

Abbreviations: y.o.=years old; N=total number of cases; M=men; W=women; SD=standard deviation; BMI=body mass index; Rz=resistance; Xc=reactance; PhA=phase angle. Data underlined are the median (min-max) of the total number of cases. Data between round brackets are mean values±SD. a, b, c, d and e indicate, respectively, young adults, adults, older adults, nonagenarians, and centenarians. The table shows the Pairwise comparisons between the different groups, *i.e*., a, b, c, d, and e stratified for gender. p-value≤0.05 is considered significant; ns=not significant.

**Figure 1.**
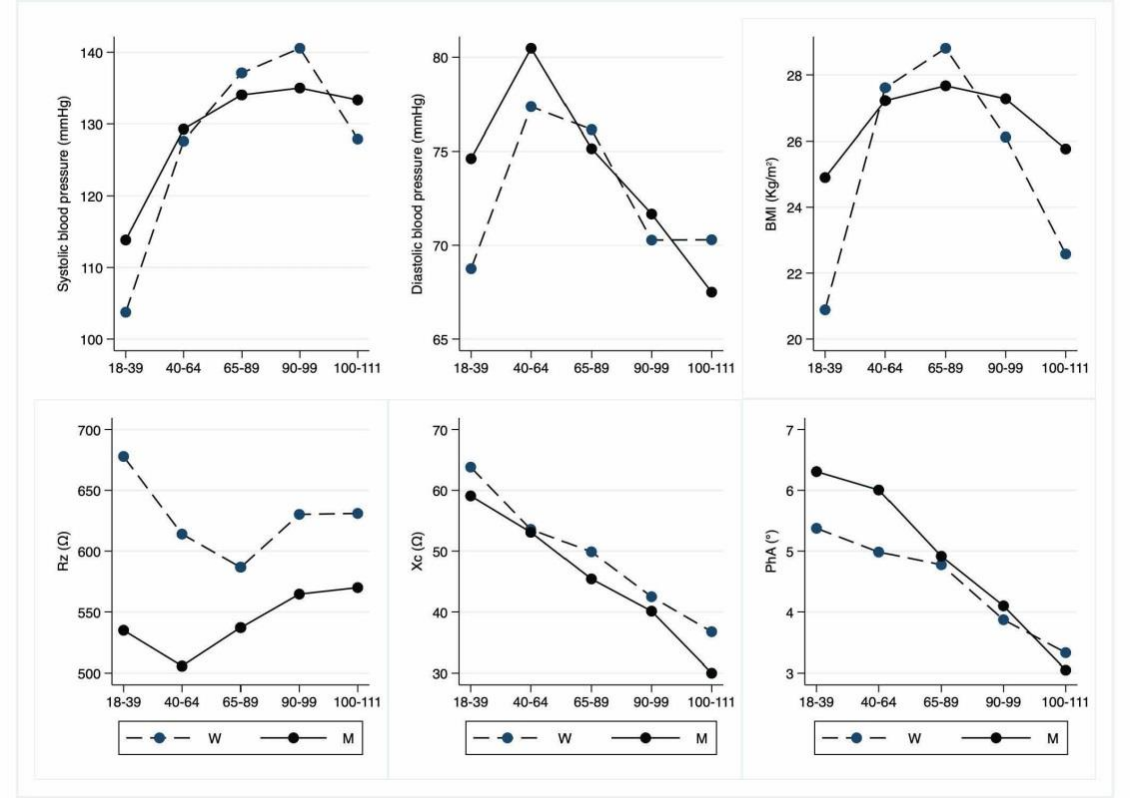
Anamnestic and bioimpedance parameters. The figure shows the trend of mean values by class of ages and gender related to anamnestic and bioimpedance parameters depicted. Y-axis reports the mean values of the analysed biomarker, x-axis reported the age-class (for the acronyms see the Table 1).[Fig F2-ad-12-7-1773]

**Table 2:** Molecular tests.

Variable	(a) Young adults (18-39 y.o.) N=29, M=13, W=16	(b) Adults (40-64 y.o.) N=40, M=21, W=19	(c) Older adults (65-89 y.o.) N=54, M=28, W=26	(d) Nonagenarians (90-99 y.o.) N=27, M=9, W=18	(e) Centenarians (100-111 y.o.) N=23, M=6, W=17	Age	Interaction		Gender
							M	W		
RTL									
	1.19 (0.91-1.80) N=29, (1.20±0.21) M=13, (1.11±0.18) W=16, (1.28±0.20)	1.04 (0.60-1.51) N=38, (1.02±0.20) M=21, (1.01±0.23) W=17, (1.05±0.16)	0.94 (0.45-1.46) N=52, (0.94±0.25) M=28, (0.91±0.25) W=24, (0.96±0.26)	0.90 (0.54-1.33) N=19, (0.97±0.25) M=8, (0.89±0.30) W=11, (0.99±0.20)	0.79 (0.46-1.60) N=20, (0.81±0.25) M=5, (0.77±0.12) W=15, (0.83±0.28)	(a vs b, c,d,e) (b vs e)	ns	(a vs c,e)	=0.04
	(a) Young adults (18-39 y.o.) N=29, M=13, W=16	(b) Adults (40-64 y.o.) N=40, M=21, W=19	(c) Older adults (65-89 y.o.) N=54, M=28, W=26	(d) Nonagenarians (90-99 y.o.) N=27, M=9, W=18	(e) Centenarians (100-111 y.o.) N=23, M=6, W=17	Age	Interaction		Gender
							M	W		
Allele number (%)									
FOXO3A rs2802292									
G	25 (43%)	33 (41%)	41 (38%)	26 (48%)	17 (37%)	ns			
T	33 (57%)	47 (59%)	67 (62%)	28 (52%)	29 (63%)				
APOE rs439358, rs7412									
ε2	5 (8%)	7 (9%)	7 (6%)	6 (11%)	6 (13%)	ns			
ε3	49 (85%)	69 (86%)	93 (87%)	47 (87%)	39 (85%)				
ε4	4 (7%)	4 (5%)	8 (7%)	1 (2%)	1 (2%)				

Abbreviations: y.o.=years old; N=total number of cases; M=men; W=women; SD=standard deviation; RTL=relative telomere length; FOXO3A=Forkhead box O 3A; APOE= Apolipoprotein-E. Data underlined are the median (min-max) of the total number of cases. Data between round brackets are mean values±SD. a, b, c, d, and e indicate, respectively, young adults, adults, older adults, nonagenarians, and centenarians. The table shows the Pairwise comparisons between the different groups, i.e., a, b, c, d, and e stratified for gender. p-value≤0.05 is considered significant; ns=not significant.

**Figure 2. F2-ad-12-7-1773:**
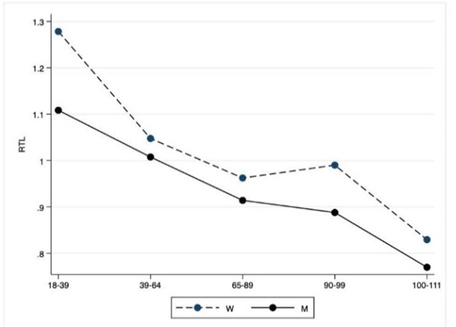
Molecular tests. The figure shows the trend of mean values by class of ages and gender related to RTL. Y-axis reports the mean values of the analysed biomarker, x-axis reported the age-class (for the acronyms see the Table 2).

The EzWayTM Direct ApoE Genotyping Kit (Komabiotech Inc) was used to analyse Apolipoprotein (Apo)E polymorphisms. The genotype was defined by the combination of three alleles ε2, ε3, and ε4. The primer mixture of ApoE genes was enabled to perform one-step multiplex ARMS-PCR. Six genotypes were analysed: ε2/ε2, ε2/ε3, ε3/ε3, ε3/ε4, ε4/ε4, and ε2/ε4. The size separation was conducted using agarose gel electrophoresis (2.5%).

### Hematological parameters

The blood sample collected in the EDTA tube of each patient was analysed at the Department of Laboratory Medicine, “P. Giaccone” University Hospital, Palermo, on a UniCel DxH 900 haematology analyser (Beckman Coulter, Inc).

### Oxidative stress parameters

The evaluation of paraoxonase (PON), Trolox equivalent antioxidant capacity (TEAC), and malondialdehyde (MDA) was conducted at the Department of Biomedical Sciences, University of Sassari, Italy, as previously described [18,19].

**Table 3:** Hematological parameters.

Variable (unit of measurement, laboratory range	(a) Young adults (18-39 y.o.) N=29, M=13, W=16	(b) Adults (40-64 y.o.) N=40, M=21, W=19	(c) Older adults (65-89 y.o.) N=54, M=28, W=26	(d) Nonagenarians (90-99 y.o.) N=27, M=9, W=18	(e) Centenarians (100-111 y.o.) N=23, M=6, W=17	Age	Interaction		Gender
							M	W		
Red blood cells (10^6^/µL, 4.2-5.5 M; 3.8-5 W)	4.84 (4.32-5.85) N=29, (4.91±0.40) M=13, (5.26±0.33) W=16, (4.63±0.19)	4.96 (3.93-6.63) N=40, (4.99±0.45) M=21, (5.15±0.46) W=19, (4.83±0.39)	4.68 (3.85-9.95) N=54, (4.86±0.88) M=28, (5.09±1.06) W=26, (4.63±0.57)	4.44 (3.41-5.36) N=27, (4.45±0.53) M=9, (4.55±0.33) W=18, (4.41±0.61)	4.38 (2.36-5.76) N=22, (4.33±0.66) M=6, (4.52±0.21) W=16, (4.27±0.77)	(a vs e) (b vs d,e)	ns	ns	<0.001
Hemoglobin (g/dL, 12-18 M; 12-16 W)	14.20 (12.00-17.00) N=29, (14.29±1.30) M=13, (15.43±0.85) W=16, (13.36±0.74)	14.55 (12.50-17.90) N=40, (14.75±1.07) M=21, (15.20±0.88) W=19, (14.25±1.08)	14.05 (11.20-17.20) N=54, (14.06±1.3) M=28, (14.73±1.05) W=26, (13.33±1.15)	12.80 (10.30-16.70) N=27, (12.93±1.53) M=9, (13.36±1.28) W=18, (12.72±1.64)	13.25 (8.30-15.20) N=22, (12.61±1.89) M=6, (13.62±1.22) W=16, (12.24±1.99)	(a vs d,e) (b vs d,e) (c vs d,e)	ns	ns	<0.001
Platelets (10^3^/µL, 150-450)	240.00 (173.00-366.00) N=29, (244.80±52.18) M=13, (238.46±48.60) W=16, (249.88±55.96)	229.50 (108.00-317.00) N=40, (221.40±46.36) M=21, (220.86±53.13) W=19, (222.05±38.98)	223.00 (102.00-371.00) N=54, (223.20±57.2) M=28, (218.39±57.56) W=26, (228.38±57.48)	204.00 (71.00-340.00) N=27, (214.40±64.87) M=9, (152.33±41.09) W=18, (245.4±50.80)	207.00 (91.00-329.00) N=22, (208.90±60.52) M=6, (191.00±54.70) W=16, (215.56±62.89)	(a vs d)	(a vs d)	ns	<0.001
Leukocytes (10^3^/µL, 4-11)	6.23 (4.28-9.41) N=29, (6.49±1.08) M=13, (6.54±1.34) W=16, (6.45±0.86)	6.57 (4.02-15.78) N=40, (6.85±2.13) M=21, (7.39±2.51) W=19, (6.26±1.46)	6.26 (3.96-13.29) N=54, (6.65±1.88) M=28, (6.75±1.78) W=26, (6.56±2.01)	6.31 (4.16-11.90) N=27, (6.80±1.94) M=9, (6.37±1.45) W=18, (7.03±2.16)	6.39 (4.21-11.33) N=22, (6.58±1.82) M=6, (7.72±2.85) W=16, (6.16±1.12)	ns	ns	ns	ns
Neutrophils (10^3^/µL, 2-8)	3.60 (1.98-5.60) N=29, (3.70±0.86) M=13, (3.47±0.84) W=16, (3.89±0.87)	3.46 (1.90-9.82) N=40, (3.93±1.49) M=21, (4.07±1.75) W=19, (3.78±1.18)	3.43 (1.97-8.7) N=54, (3.76±1.26) M=28, (3.96±1.46) W=26, (3.55±0.99)	3.64 (1.5-8.43) N=27, (4.12±1.57) M=9, (3.62±0.57) W=18, (4.39±1.86)	4.13 (2.44-7.57) N=22, (4.04±1.22) M=6, (4.63±1.84) W=16, (3.83±0.88)	ns	ns	ns	ns
Lymphocytes (10^3^/µL, 1-5)	2.00 (1.31-3.46) N=29, (2.08±0.55) M=13, (2.29±0.67) W=16, (1.92±0.39)	2.02 (1.11-5.03) N=40, (2.16±0.7) M=21, (2.46±0.80) W=19, (1.83±0.36)	1.96 (0.77-6.18) N=54, (2.08±0.88) M=28, (1.95±0.61) W=26, (2.23±1.10)	1.75 (0.60-4.14) N=27, (2.00±0.85) M=9, (1.76±0.61) W=18, (2.13±0.94)	1.58 (0.84-3.44) N=22, (1.74±0.63) M=6, (2.00±0.94) W=16, (1.64±0.48)	ns	ns	ns	ns
N/L	1.78 (0.97-3.61) N=29, (1.89±0.70) M=13, (1.62±0.64) W=16, (2.12±0.69)	1.79 (0.95-3.54) N=40, (1.88±0.62) M=21, (1.69±0.62) W=19, (2.08±0.57)	1.82 (0.94-5.22) N=54, (2.01±0.89) M=28, (2.23±1.04) W=26, (1.77±0.63)	1.93 (0.75-12.83) N=27, (2.53±2.25) M=9, (2.23±0.73) W=18, (2.69±2.73)	2.45 (1.01-5.22) N=22, (2.54±0.98) M=6, (2.63±1.423) W=16, (2.51±0.83)	ns	ns	ns	ns

Abbreviations: y.o.= years old; N=total number of cases; M=men; W=women; SD=standard deviation; N/L=Neutrophils/lymphocytes ratio. Data underlined are the median (min-max) of the total number of cases. Data between round brackets are mean values±SD. a, b, c, d, and e indicate, respectively, young adults, adults, older adults, nonagenarians, and centenarians. The table shows the Pairwise comparisons between the different groups, *i.e*., a, b, c, d, and e stratified for gender. p-value≤0.05 is considered significant; ns=not significant.

### Hematochemical parameters

The tests were carried out at the Department of Laboratory Medicine, “P. Giaccone” University Hospital, Palermo, according to standard procedures. C-reactive protein (CRP) measurement was performed by immunoturbidimetry methods, uric acid (UA) by colorimetric test, and lipid parameters by enzymatic colorimetric test through Roche/Hitachi Cobas system.

The concentration of serum low density lipoprotein (LDL) was calculated by using the Friedewald equation: LDL= [total cholesterol-high(H)DL-(triglycerides/5)].

Insulin resistance status was assessed as homeostasis model assessment of insulin resistance (HOMA Index) according to the following formula: [insulin (μU/mL)* glycaemia (mg/dL)]/405. Vitamin D was evaluated as previously described [20].

### Statistical analysis

Data are presented both as median, minimum, maximum values, and mean±standard deviation (SD). For continuous variables, the two ways Analysis of Variance (ANOVA) is used to test if gender, age or both of them as joint events (*i.e*., their interaction), produce an effect on the quantitative parameters. The Fisher test is considered to evaluate the significance of the results. For each statistically significant effect, we conduct a *post-hoc* multiple comparison test using Bonferroni method. For categorical variables, we consider the chi-squared test or the Fisher exact test to compare differences between groups of age and for gender. All analyses were performed using Stata version 14.2 and all hypothesis testing are considered statistically significant for *p*≤ 0.05.

## RESULTS

### Anamnestic and bioimpedance parameters

Table 4 reports blood pressure, sleeping habits, anthropometric features (BMI), and BIA parameters, while the other anamnestic and clinical data reported below, especially about LLIs description, are not tabulated. Figure 1 depicts graphically the blood pressure, BMI, and BIA parameters according to classes of age.

As expected, systolic but not diastolic blood pressure is increased in the adult group compared to that observed in young adults. No significant difference in sleep habits (hours/night) is observed between the groups [21].

All LLIs are no smokers with a small percentage of former smokers (20%). The percentage of smokers is quite high in young adults and adults (32%). Concerning MMSE, it is noteworthy that the mean values +SD of LLIs group suggest moderate to severe dementia (non-agenarians 18.10±5.8; centenarians 17.00±6.1), with no significant gender differences. Similar MMSE values (16.45+4.4) were obtained in a study conducted in a group of New York centenarians [22].

However, it must be considered that values are lower in people with poor levels of education and with compromised sense organs, in particular sight and hearing (data not shown). The mean GDS±SD score indicates that most nonagenarians (5.7±3.1) and centenarians (4.8±4.4) are not depressed or mildly depressed, with no significant gender differences. In a recent study performed in another South European population, the prevalence of depression in centenarians was 35% [23].

Concerning ADL (*e.g*., personal hygiene, dressing, toileting/continence, ambulating, and eating), most LLIs have a good degree of independence. Conversely, regarding IADL (*e.g*., food preparation, financial administration, housekeeping, use of telephone, and responsibility for own medication) almost all centenarians show a decrease in performing these activities in the last years before the interview. Again, these observations agree to Jopp *et al*. [22].

About eating habits, at the time of interview the diet of LLIs was not strictly adherent to Mediterranean pattern for some food choices (*e.g*., frequent consumption of sweets, sugar, eggs, and biscuits as well as no consumption of whole grain). Nonetheless, they partially followed the Mediterranean diet (MedDiet) when compared to the younger subjects and, during their childhood and adolescence. Indeed, at present LLIs often ate cereals, like pasta, extra virgin olive oil (EVOO), milk, fruits, and legumes, very rare assuming red and cured meat, and more frequently choosing white meat (like chicken) and bluefish. Instead, the younger participants showed low consumption of vegetables, high assumption of sweets and potatoes, and ate more red and cured meat, but their diet is quite rich in fruit, legumes, and EVOO.

Regarding drug assumption, 71% of nonagenarians and 52% of centenarians are treated with anti-hypertensive, diuretics, and antiplatelet agents, whereas 18% of nonagenarians and 39% of centenarians are treated with anxiolytic ones. Sight or hearing deficits are present in 64% of nonagenarians and 70% of centenarians.

Moreover, we observed a significant increase of BMI in all groups, except for centenarians when compared to values observed in young adults.

Interestingly, the centenarian BMI values are significantly lower than values observed in older adults with no gender effect.

Lastly, the resistance values obtained from BIA are not significantly different between the various groups, including centenarians, except for young and older female adults. The reactance values are significantly decreased in all classes of age, including centenarians, when compared to values of young people. Centenarian measurements are also significantly lower than those observed in the remaining classes of age. The PhA parameters decrease in all older classes of age, *i.e*., older adults, nonagenarians, and centenarians when compared to young adults and adults, both on the whole and separately in men and women. Resistance, reactance and PhA are significantly different between male and female cohorts.

### Molecular tests

**Figure 3.**
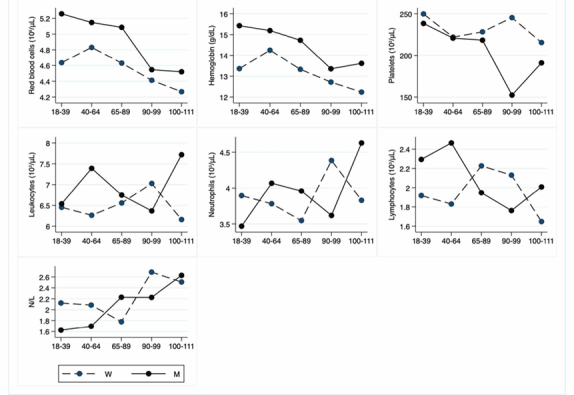
Hematological parameters. The figure shows the trend of mean values by class of ages and gender related to haematological parameters. Y-axis reports the mean values of the analysed biomarker, x-axis reported the age-class (for the acronyms see the Table 3).

**Table 4:** Oxidative stress parameters.

Variable (unit of measurement)	(a) Young adults (18-39 y.o.) N=29, M=13, W=16	(b) Adults (40-64 y.o.) N=40, M=21, W=19	(c) Older adults (65-89 y.o.) N=54, M=28, W=26	(d) Nonagenarians (90-99 y.o.) N=27, M=9, W=18	(e) Centenarians (100-111 y.o.) N=23, M=6, W=17	Age	Interaction		Gender
							M	W		
PON (U/L)	114.50 (40.60-392.40) N=29, (133.54±82.98) M=13, (114.60±62.40) W=16, (148.90±95.80)	117.30 (26.78-312.10) N=39, (119.37±64.10) M=21, (115.11±58.51) W=18, (124.30±71.50)	89.05 (19.40-300.10) N=52, (106.99±64.01) M=28, (117.60±63.64) W=24, (94.00±63.53)	59.56 (21.20-132.00) N=26, (69.07±34.10) M=8, (72.71±38.10) W=18, (67.45±33.22)	75.74 (43.40-254.80) N=20 (91.97±55.95) M=5, (93.64±63.80) W=15, (91.40±55.53)	(a vs d) (b vs d)	ns	ns	ns
TEAC (mM)	3601.20 (2589.60-4722) N=29, (3563.36±398.58) M=13, (3526.70±490.70) W=16, (3593.20±319.00)	3879.60 (1805.70-5574) N=39, (4016.25±781) M=21, (3979.63±818.88) W=18, (4058.96±755.61)	4136.50 (2819.20-5572) N=52, (4127.14±691.30) M=28, (4027.08±679.07) W=24, (4243.90±701.4)	5042.30 (3710.70-5653) N=26, (4921.37±452.95) M=8, (4659.93±556.56) W=18, (5037.57±357.35)	4309.10 (2967.70-5774) N=20, (4246.99±713.19) M=5, (4541.38±1004.34) W=15, (4148.90±600.70)	(a vs b,c,d,e) (b vs d) (c vs d)	ns	ns	ns
MDA (µmol/L)	2.60 (1.19-5.40) N=29, (2.78±1.025) M=13, (2.96±0.82) W=16, (2.64±1.20)	2.70 (0.90-4.50) N=39, (2.71±0.91) M=21, (2.84±0.76) W=18, (2.57±1.06)	2.51 (0.60-6.40) N=52, (2.54±1.09) M=28, (2.86±1.21) W=24, (2.20±0.80)	1.91 (0.98-4.25) N=26, (2.09±0.87) M=8, (2.30±1.03) W=18, (2.00±0.80)	2.18 (1.01-5.80) N=20, (2.43±1.14) M=5, (2.75±0.64) W=15, (2.32±1.26)	ns	ns	ns	0.03

Abbreviations: y.o.=years old; N=total number of cases; M=men; W=women; SD=standard deviation; PON=paraoxonase; TEAC=trolox equivalent antioxidant capacity; MDA=malondialdehyde. Data underlined are the median (min-max) of the total number of cases. Data between round brackets are mean values±SD. a, b, c, d, and e indicate, respectively, young adults, adults, older adults, nonagenarians, and centenarians. The table shows the Pairwise comparisons between the different groups, *i.e*., a, b, c, d, and e stratified for gender. p-value≤0.05 is considered significant; ns=not significant.

Concerning the allelic frequencies of FOXO3A G and T SNPs and APOE genotypes, no significant differences are observed between the various classes of age on the whole and when analysed according to gender. However, it is noteworthy the lower, although not significant, number of events related to ε4 allele in nonagenarians and centenarians when compared to other age groups.

### Hematological parameters

Table 3 depicts the values of red blood cells, hemoglobin, platelets, leukocytes, neutrophils, lymphocytes, and their ratio (neutrophils/lymphocytes, N/L). Figure 3 depicts graphically hematological parameters, according to classes of age.

As expected, the values of red blood cells, hemoglobin and platelets are significantly different between men and women [24,25]. Both hemoglobin and red blood cells values are lower in LLIs when compared to other age classes. The platelets also decrease with age, but significance is only obtained comparing male young adults to nonagenarians. However, it is noteworthy the not significant increase of N/L ratio in older people compared to younger [26]. No significant differences are observed in the other parameters under study.

Comparing to Japanese centenarians recruited over the past 20 years, the values of red blood cells, hemoglobin, leukocytes and platelets were respectively 3.58+0.52, 5.7+0.7, 5.4+1.5 and 188+61, thus lower of the means of our centenarians but in the range [27].

**Figure 4.**
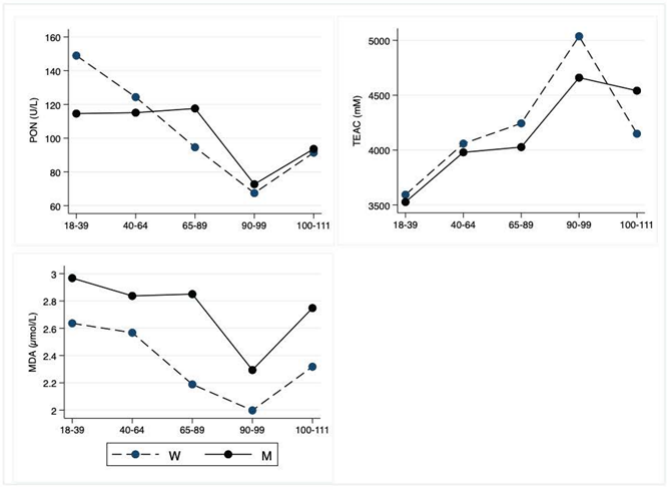
Oxidative Stress Parameters. The figure shows the trend of mean values by class of ages and gender related to oxidative parameters. Y-axis reports the mean values of the analysed biomarker, x-axis reported the age-class (for the acronyms see the Table 4).

**Table 5:** Hematochemical parameters: Endocrine parameters.

Variable (unit of measurement)	(a) Young Adults (18-39 y.o.) N=29; M=13; W=16	(b) Adults (40-64 y.o.) N=40; M=21; W=19	(c) Older adults (65-89 y.o.) N=54; M=28; W=26	(d) Nonagenarians (90-99) y.o. N=27; M=9; W=18	(e) Centenarians (100-111) y.o. N=23; M=6; W=17	Age	Interaction		Gender
							M	W		
TSH (µIU/mL, 0.27-4,2)	2.07 (0.59-6.06) N=29, (2.21±1.09) M=13, (2.47±1.23) W=16, (2.00±0.95)	1.74 (0.31-4.67) N=40, (1.88±0.89) M=21, (2.05±1.02) W=19, (1.70±0.71)	1.69 (0.39-7.72) N=54, (2.05±1.40) M=28, (1.94±1.28) W=25, (2.17±1.53)	1.50 (0.01-9.94) N=26, (2.08±2.30) M=9, (2.29±2.47) W=17, (1.96±2.27)	1.70 (0.73-10.70) N=23, (2.53±2.15) M=6, (1.92±1.05) W=17, (2.75±2.41)	ns	ns	ns	ns
FT3 (pg/mL, 2-4.4)	3.09 (2.21-3.85) N=29, (3.15±0.40) M=13, (3.25±0.41) W=16, (3.08±0.39)	3.12 (2.40-4.27) N=40, (3.20±0.38) M=21, (3.28±0.32) W=19, (3.11±0.42)	3.03 (2.19-3.69) N=54, (3.04±0.32) M=28, (3.06±0.28) W=26, (3.03±0.37)	2.80 (1.80-4.08) N=27, (2.76±0.56) M=9, (2.85±0.58) W=18, (2.72±0.57)	2.54 (1.90-3.58) N=23, (2.58±0.46) M=6, (2.73±0.22) W=17, (2.52±0.51)	(a vs d,e) (b vs d,e) (c vs e)	ns	ns	=0.04
FT4 (ng/dL, 0.93-1.7)	1.28 (0.98-1.65) N=29, (1.30±0.16) M=13, (1.37±0.18) W=16, (1.24±0.13)	1.17 (0.94-1.68) N=36, (1.20±0.17) M=19, (1.16±0.16) W=17, (1.25±0.17)	1.15 (0.77-1.80) N=52, (1.16±0.20) M=27, (1.15±0.21) W=25, (1.17±0.19)	1.18 (0.88-1.89) N=25, (1.24±0.23) M=9, (1.13±0.20) W=16, (1.30±0.23)	1.17 (0.94-1.56) N=20, (1.21±0.17) M=4, (1.30±0.26) W=16, (1.19±0.14)	(a vs c)	(a vs c)	ns	ns
Insulin (µU/mL, 2.6-24.9)	5.66 (2.48-15.40) N=29, (6.98±3.44) M=13, (7.49±3.80) W=16, (6.57±3.19)	9.23 (3.05-36.10) N=40, (10.49±6.97) M=21, (11.81±8.52) W=19, (9.04±4.51)	9.71 (3.80-49.40) N=54, (14.68±14.94) M=27, (14.68±17.42) W=26, (14.69±12.05)	6.22 (1.33-15.70) N=27, (9.65±15.73) M=9, (6.65±3.01) W=17, (11.14±19.15)	5.04 (2.50-23.10) N=23, (7.40±5.80) M=6, (9.32±7.19) W=17, (6.73±5.31)	(a vs c)	ns	ns	ns
HOMA index (0.23-2.5)	1.20 (0.47-3.19) N=29, (1.42±0.77) M=13, (1.57±0.88) W=16, (1.31±0.68)	2.06 (0.55-13.55) N=40, (2.55±2.43) M=21, (3.04±3.09) W=19, (2.01±1.24)	2.20 (0.80-38.83) N=53, (3.86±5.62) M=28, (4.06±6.97) W=25, (3.64±3.69)	1.32 (0.25-25.02) N=27, (2.30±4.61) M=9, (1.56±1.01) W=18, (2.67±5.62)	1.09 (0.53-5.76) N=23, (1.69±1.46) M=6, (2.14±1.85) W=17, (1.53±1.33)	ns	ns	ns	ns
Glycaemia (mg/dL, 70-100)	81 (65-98) N=29, (81.07±6.63) M=13, (82.69±8.29) W=16, (79.75±4.78)	88 (74-152) N=40, (91.43±16.14) M=21, (95.52±18.90) W=19, (86.89±11.21)	93.5 (63-207) N=54, (98.24±21.71) M=28, (98.64±16.12) W=26, (97.81±26.80)	84 (65-147) N=27, (86.78±16.00) M=9, (90.33±21.90) W=18, (85±12.48)	89 (72-108) N=23, (89.13±9.25) M=6, (89.83±6.62) W=17, (88.88±10.19)	(a vs c)	ns	ns	ns

Abbreviations: y.o.=years old; N=total number of cases; M=men; W=women; SD=standard deviation; TSH=thyroid-stimulating hormone; FT3=free triiodothyronine; FT4=free thyroxine; HOMA=homeostasis model assessment. HOMA index was calculated by the following formula: [insulin (μU/mL)*glycaemia (mg/dL)]/405. Data underlined are the median (min-max) of the total number of cases. Data between round brackets are mean values±SD. a, b, c, d, and e indicate, respectively, young adults, adults, older adults, nonagenarians, and centenarians. The table shows the Pairwise comparisons between the different groups, *i.e*., a, b, c, d, and e stratified for gender. p-value≤0.05 is considered significant; ns=not significant.

**Figure 5.**
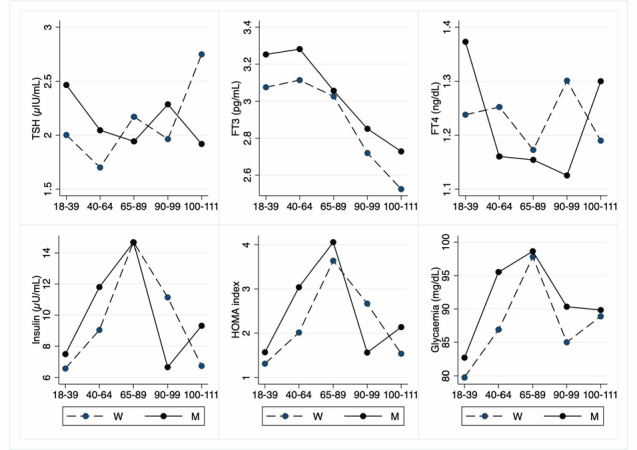
Hematochemical parameters: Endocrine parameters. The figure shows the trend of mean values by class of ages and gender related to endocrine parameters reports the mean values of the analysed biomarker, x-axis reported the age-class (for the acronyms see the table 5).

**Table 6:** Hematochemical parameters: Liver parameters.

Variable (unit of measurement)	(a) Young Adults (18-39 y.o.) N=29; M=13; W=16	(b) Adults (40-64 y.o.) N=40; M=21; W=19	(c) Older adults (65-89 y.o.) N=54; M=28; W=26	(d) Nonagenarians (90-99) y.o. N=27; M=9; W=18	(e) Centenarians (100-111) y.o. N=23; M=6; W=17	Age	Interaction		Gender
							M	W		
ALT (U/L, <41)	13.00 (7.00 -27.00) N=29, (14.48±5.42) M=13, (17.54±6.37) W=16, (12.00±2.78)	17.00 (4.00 -72.00) N=40, (23.18±16.09) M=21, (29.76±19.31) W=19, (15.89±6.31)	15.50 (7.00 -55.00) N=54, (18.54±10.02) M=28, (17.43±7.21) W=26, (19.73±12.41)	11.00 (3.00 -33.00) N=27, (11.52±6.47) M=9, (13.67±7.92) W=18, (10.44±5.55)	9.00 (5.00 -15.00) N=23, (9.74±2.77) M=6, (12.67±2.42) W=17, (8.70±2.08)	(a vs b) (b vs d,e) (c vs e)	(a vs b) (b vs c,d,e)	(c vs e)	0.03
AST (U/L, <40)	16.00 (12.00 -23.00) N=29, (16.17±2.93) M=13, (17.23±3.37) W=16, (15.31±2.27)	17.00 (11.00 -35.00) N=40, (19.68±6.58) M=21, (21.57±6.79) W=19, (17.58±5.81)	17.50 (11.00 -35.00) N=54, (19.85±10.03) M=28, (17.79±4.24) W=26, (22.08±13.56)	15.00 (6.00 -33.00) N=27, (15.67±5.54) M=9, (15.00±4.80) W=18, (16.00±5.98)	16.00 (10.00 -28.00) N=23, (17.61±4.62) M=6, (19.17±4.49) W=17, (17.06±4.67)	ns	ns	ns	ns
GGT (U/L, 8-61)	11.00 (5.00 -45.00) N=29, (14.07±9.47) M=13, (16.15±10.06) W=16, (12.38±8.92)	17.50 (7.00 -112.00) N=40, (28.25±26.49) M=21, (36.67±28.56) W=19, (18.95±20.97)	17.50 (7.00 -213.00) N=54, (26.09±33.23) M=28, (29.14±38.93) W=26, (22.81±8.96)	10.00 (5.00 -43.00) N=27, (13.59±8.66) M=9, (15.00±8.37) W=18, (12.89±8.96)	14.00 (6.00 -68.00) N=23, (16.78±14.88) M=6, (33.30±22.11) W=17, (10.94±3.53)	ns	ns	ns	0.01
Bilirubin (mg/dL, <1.20)	0.86 (0.28-2.33) N=29, (0.95±0.52) M=13, (1.08±0.57) W=16, (0.85±0.47)	0.51 (0.26-2.40) N=40, (0.68±0.48) M=21, (0.83±0.56) W= 19, (0.51±0.32)	0.52 (0.17-1.71) N=54, (0.53±0.25) M=28, (0.63±0.29) W=26, (0.43±0.14)	0.44 (0.16-0.95) N=27, (0.46±0.20) M=9, (0.49±0.20) W=18, (0.44±0.20)	0.40 (0.20-1.78) N=23, (0.55±0.40) M=6, (0.91 0.65) W=17, (0.43±0.15)	(a vs b,c,d)	ns	ns	<0.001
Conjugated bilirubin (mg/dL, <0.30)	0.35 (0.16-0.65) N=29, (0.36±0.13) M=13, (0.39±0.13) W=16, (0.33±0.13)	0.22 (0.12-0.62) N=40, (0.25±0.12) M=21, (0.29±0.12) W=19, (0.21±0.10)	0.21 (0.11-0.49) N=54, (0.23±0.08) M=28, (0.26±0.09) W=26, (0.18±0.05)	0.20 (0.10-0.45) N=27, (0.21±0.08) M=9, (0.23±0.10) W=18, (0.20±0.07)	0.19 (0.12-0.95) N=23, (0.24±0.18) M=6, (0.41±0.29) W=17, (0.18±0.04)	(a vs b,c,d)	(a vs c,d)	(a vs b,c,d,e)	<0.001
Albumin (g/L, 38-48)	46.05 (33.90-54.40) N=28, (46.13±3.81) M=12, (48.06±3.02) W=16, (44.68±3.77)	45.30 (40.40-50.60) N=39, (45.42±2.92) M=21, (46.48±2.47) W=18, (44.18±2.98)	43.00 (38.40-51.60) N=53, (43.45±2.84) M=28, (43.96±3.06) W=26, (42.88±2.50)	39.10 (34.20-47.50) N=27, (39.66 3.48) M=9, (40.18±3.53) W=18, (39.40±3.53)	39.30 (31.10-46.40) N=22, (39.02±3.92) M=5, (40.16±2.73) W=17, (38.69±4.22)	(a vs c,d,e) (b vs d,e) (c vs d,e)	ns	ns	<0.001
Total proteins (g/L, 66-87)	71.50 (59.90-82.20) N=29, (71.52±5.78) M=13, (73.01±4.58) W=16, (70.31±6.50)	69.95 (59.60-82.00) N=40, (69.75±4.54) M=21, (69.93±2.62) W=19, (69.55±6.09)	68.10 (60.50-78.10) N=54, (68.32±4.25) M=28, (68.19±4.37) W=26, (68.46±4.20)	67.70 (54.30-79.00) N=27, (68.01±5.48) M=9, (68.47±3.94) W=18, (67.78±6.21)	67.00 (58.50-80.00) N=23, (66.73±5.28) M=6, (67.10±6.56) W=17, (66.61±4.98)	(a vs c,e)	ns	ns	ns

Abbreviations: y.o.=years old; N=total number of cases; M=men; W=women; SD=standard deviation; ALT=alanine transaminase; AST=aspartate transaminase; GGT=gamma-glutamil transferase. Data underlined are the median (min-max) of the total number of cases. Data between round brackets are mean values±SD. a, b, c, d, and e indicate, respectively, young adults, adults, older adults, nonagenarians, and centenarians. The table shows the Pairwise comparisons between the different groups, *i.e*., a, b, c, d, and e stratified for gender. p-value≤0.05 is considered significant; ns=not significant.

**Figure 6.**
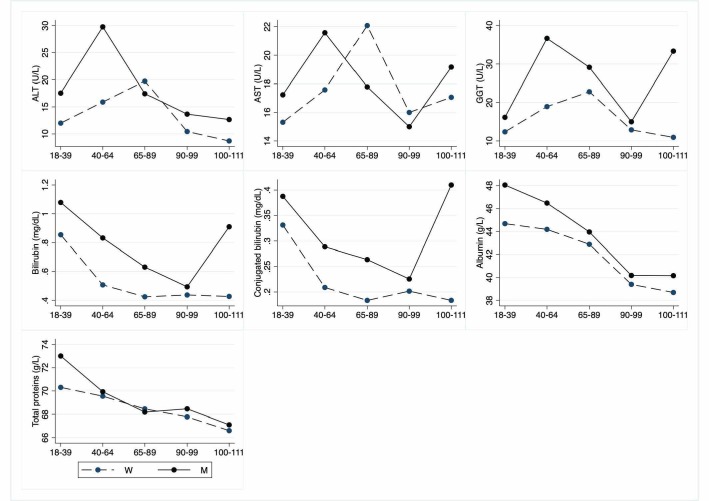
Hematochemical parameters: Liver parameters. The figure shows the trend of mean values by class of ages and gender related to liver parameters. Y-axis reports the mean values of the analysed biomarker, x-axis reported the age-class (for the acronyms see the table 6).

**Table 7:** Hematochemical parameters: Iron parameters.

Variable (unit of measurement)	(a) Young Adults (18-39 y.o.) N=29; M=13; W=16	(b) Adults (40-64 y.o.) N=40; M=21; W=19	(c) Older adults (65-89 y.o.) N=54; M=28; W=26	(d) Nonagenarians (90-99) y.o. N=27; M=9; W=18	(e) Centenarians (100-111) y.o. N=23; M=6; W=17	Age	Interaction		Gender
							M	W		
Iron (µg/dL, 37-145)	82.00 (23.00-209.00) N=29, (92.86±37.62) M=13, (100.08±26.96) W=16, (87.00±44.48)	99.00 (31.00-175.00) N=40, (96.13±33.46) M=21, (101.33±32.98) W=19, (90.37±33.93)	89.50 (43.00-139.00) N=54, (87.85±22.39) M=28, (95.25±20.82) W=26, (79.88±21.61)	62.00 (22.00-151.00) N=27, (69.19±28.70) M=9, (68.00±26.54) W=18, (66.78±30.46)	65.00 (25.00-117.00) N=23, (66.17±25.25) M=6, (67.83±29.42) W=17, (65.59±24.58)	(a vs d,e) (b vs d,e)	ns	ns	ns
Transferrin (mg/dL, 200-360)	250.00 (151.00-320.00) N=29, (247.52±34.87) M=13, (239.92±30.38) W=16, (253.69±37.96)	246.00 (184.00-324.00) N=39, (248.64±31.90) M=21, (249.67±34.31) W=18, (247.44±29.78)	247.00 (197.00-329.00) N=53, (246.98±29.87) M=27, (245.81±32.71) W=26, (248.19±27.19)	243.00 (143.00-322.00) N=26, (246.38±44.18) M=8, (250.50±52.64) W=18, (244.56±41.45)	236.00 (171.00-269.00) N=23, (229.38±32.76) M=6, (243.00±28.22) W=17, (224.53±33.65)	ns	ns	ns	ns
Ferritin (ng/mL, 15-400)	42.00 (5.00-327.00) N=29, (89.66±96.78) M=13, (131.75±85.97) W=16, (37.31±19.00)	106.00 (17.00-580.00) N=40, (147.23±124.79) M=21, (191.48±142.86) W=19, (98.32±79.07)	116.50 (35.00-451.00) N=54, (134.31±85.99) M=28, (168.85±103.58) W=26, (97.12±36.25)	85.00 (14.00-447.00) N=27, (122.93±108.10) M=9, (110.22±114.31) W=18, (129.28±107.68)	69.00 (12.00-565.00) N=23, (137.87±144.81) M=6, (194.83±197.00) W=17, (117.76±122.72)	ns	ns	ns	<0.001

Abbreviations: y.o.=years old; N=total number of cases; M=men; W=women; SD=standard deviation. Data underlined are the median (min-max) of the total number of cases. Data between round brackets are mean values±SD. a, b, c, d, and e indicate, respectively, young adults, adults, older adults, nonagenarians, and centenarians. The table shows the Pairwise comparisons between the different groups, *i.e*., a, b, c, d, and e stratified for gender. p-value≤0.05 is considered significant; ns=not significant.

**Figure 7.**
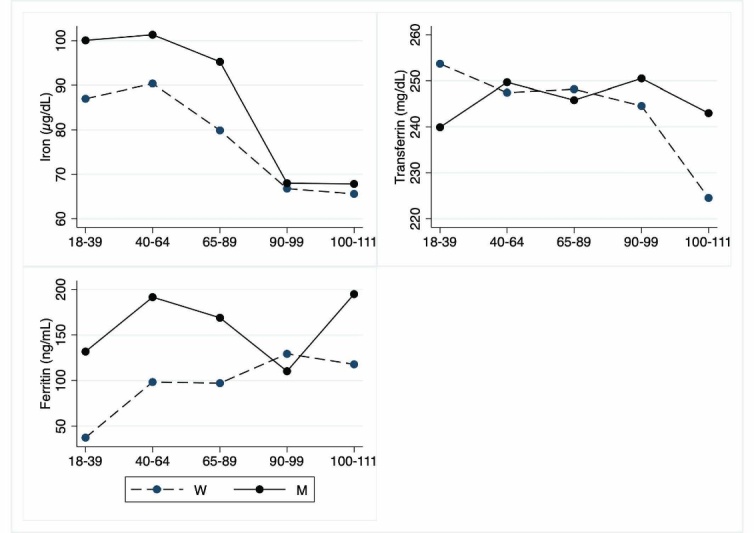
Hematochemical parameters: Iron parameters. The figure shows the trend of mean values by class of ages and gender related to iron parameters. Y-axis reports the mean values of the analysed biomarker, x-axis reported the age-class (for the acronyms see the table 7).

**Figure 8.**
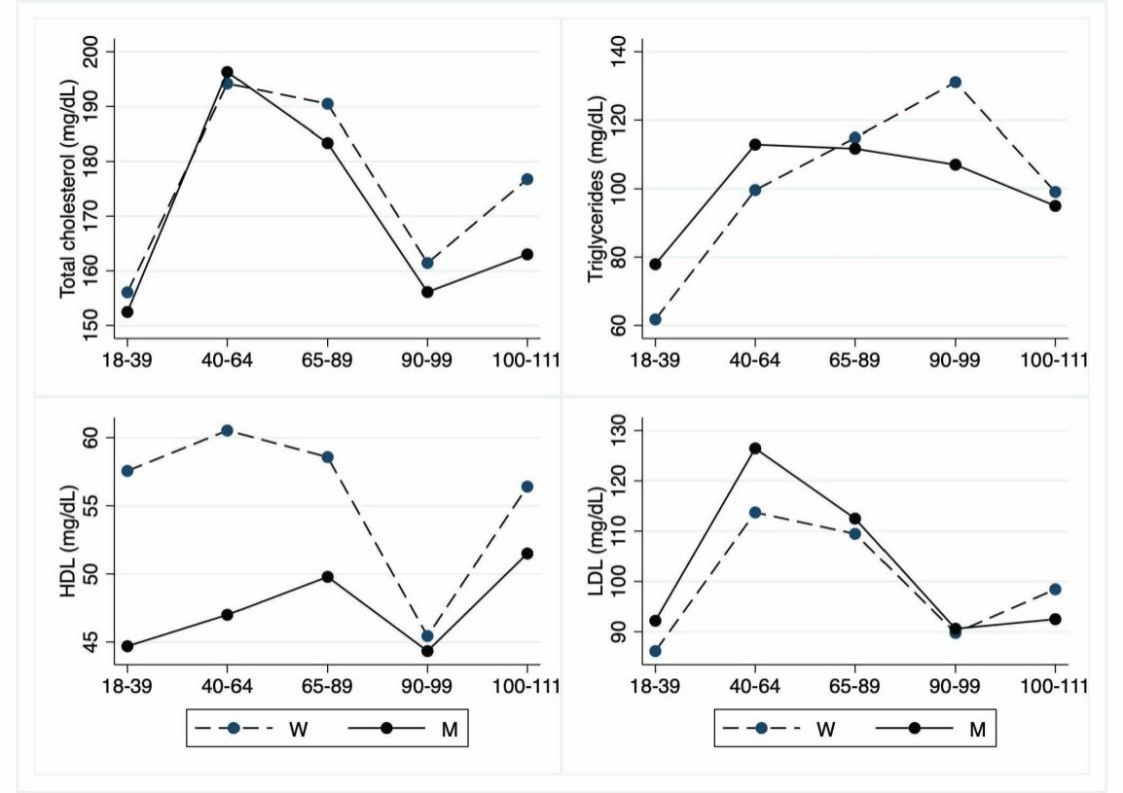
Hematochemical parameters: Lipid parameters. The figure shows the trend of mean values by class of ages and gender related to lipid parameters. Y-axis reports the mean values of the analysed biomarker, x-axis reported the age-class (for the acronyms see the table 8).

**Table 8:** Hematochemical parameters: Lipid parameters.

Variable (unit of measurement)	(a) Young Adults (18-39 y.o.) N=29; M=13; W=16	(b) Adults (40-64 y.o.) N=40; M=21; W=19	(c) Older adults (65-89 y.o.) N=54; M=28; W=26	(d) Nonagenarians (90-99 y.o.) N=27; M=9; W=18	(e) Centenarians (100-111 y.o.) N=23; M=6; W=17	Age	Interaction		Gender
							M	W		
Total cholesterol (mg/dL <200)	154.00 (102.00-213.00) N=29, (154.40±26.76) M=13, (152.46±29.97) W=16, (156.06±24.74)	195.00 (155.00-243.00) N=40, (195.30±25.38) M=21, (196.28±25.69) W=19, (194.18±25.69)	189.00 (110.00-252.00) N=54, (186.80±28.28) M=28, (183.35±31.13) W=26, (190.48±24.93)	153.00 (103.00-230.00) N=27, (159.70±32.31) M=9, (156.10±33.13) W=18, (161.43±32.71)	168.00 (127.00-248.00) N=23, (173.20±29.37) M=6, (163.00±30.63) W=17, (176.76±28.99)	(a vs b,c)	ns	ns	ns
Triglycerides (mg/dL, <200)	61.00 (20.00-146.00) N=29, (69.00±34.70) M=13, (77.92±41.10) W=16, (61.75±27.76)	94.00 (39.00-232.00) N=40, (106.60±51.31) M=21, (112.95±49.83) W=19, (99.65±53.35)	96.50 (54.00-215.00) N=54, (113.30±45.53) M=28, (111.74±46.95) W=26, (114.89±44.82)	113.00 (52.00-281.00) N=27, (123.10±59.00) M=9, (107.07±59.25) W=18, (131.08±58.89)	97.00 (53.00-167.00) N=23, (98.02±28.79) M=6, (95.00±42.87) W=17, (99.08±23.68)	(a vs b,c,d)	ns	ns	ns
HDL (mg/dL, >40M; >50F)	51.00 (36.00-70.00) N=29, (51.79±10.61) M=13, (44.69±7.49) W=16, (57.56±9.29)	50.50 (28.00-116.00) N=40, (53.43±15.63) M=21, (47.00±7.70) W=19, (60.53±19.02)	54.00 (32.00-91.00) N=54, (54.02±12.75) M=28, (49.79±11.73) W=26, (58.58±12.42)	45.00 (27.00-71.00) N=27, (45.07±960) M=9, (44.33±6.84) W=18, (45.44±10.90)	54.00 (36.00-79.00) N=23, (55.13±13.34) M=6, (51.50±10.65) W=17, (56.41±14.23)	(b vs d) (c vs d)	ns	ns	ns
LDL (mg/dL,70-129)	85.40 (51.40-144.80) N=29, (88.86±21.53) M=13, (92.18±26.61) W=16, (86.15±16.77)	125.60 (74.60-162.80) N=40, (120.40±22.08) M=21, (126.46±21.06) W=19, (113.72±21.75)	109.00 (54.00-176.60) N=54, (111.00±26.92) M=28, (112.50±28.06) W=26, (109.46±26.09)	84.80 (43.60-151.00) N=27, (90.05±27.84) M=9, (90.60±28.50) W=18, (89.77±28.33)	94.30 (55.20-177.60) N=22, (96.82±27.02) M=6, (92.50±24.50) W=16, (98.44±28.49)	(a vs b,c) (b vs d,e) (c vs d)	ns	ns	ns

Abbreviations: y.o.=years old; N=total number of cases; M=men; W=women; SD=standard deviation; HDL=high density lipoprotein; LDL=low density lipoprotein. Data underlined are the median (min-max) of the total number of cases. Data between round brackets are mean values±SD. a, b, c, d, and e indicate, respectively, young adults, adults, older adults, nonagenarians, and centenarians. The table shows the Pairwise comparisons between the different groups, *i.e*., a, b, c, d, and e stratified for gender. p-value≤0.05 is considered significant; ns=not significant.

Comparing to Japanese centenarians recruited over the past 20 years, the values of red blood cells, hemoglobin, leukocytes and platelets were respectively 3.58+0.52, 5.7+0.7, 5.4+1.5 and 188+61, thus lower of the means of our centenarians but in the range [27].

### Oxidative stress parameters

In Table 4 are compared the mean values of PON, TEAC, and MDA. Figure 4 depicts graphically oxidative stress parameters, according to classes of age.

PON significantly decreases from young to nonagenarians, with a not statistically significant decrease in centenarians. TEAC significantly increases from young to LLIs, although the values slightly decrease in centenarians in comparison to nonagenarians. The MDA does not show significant age-related differences but a significant heterogeneity between men and women.

### Hematochemical parameters

**Figure 9.**
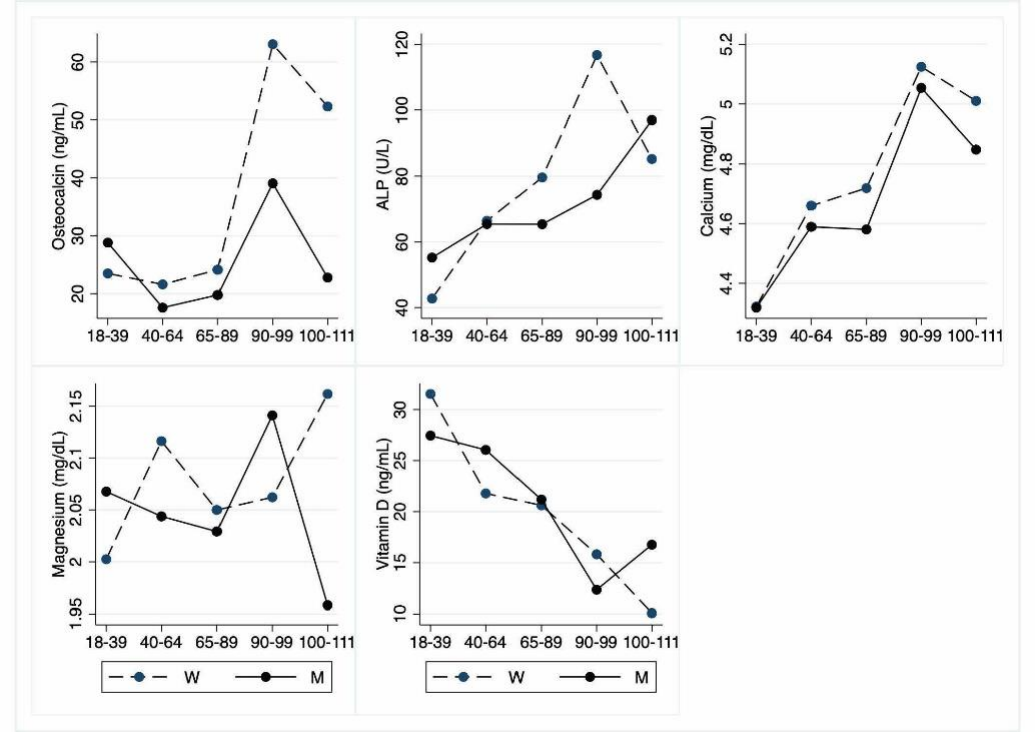
Hematochemical parameters: Bone parameters. The figure shows the trend of mean values by class of ages and gender related to bone parameters. Y-axis reports the mean values of the analysed biomarker, x-axis reported the age-class (for the acronyms see the table 9).

**Figure 10.**
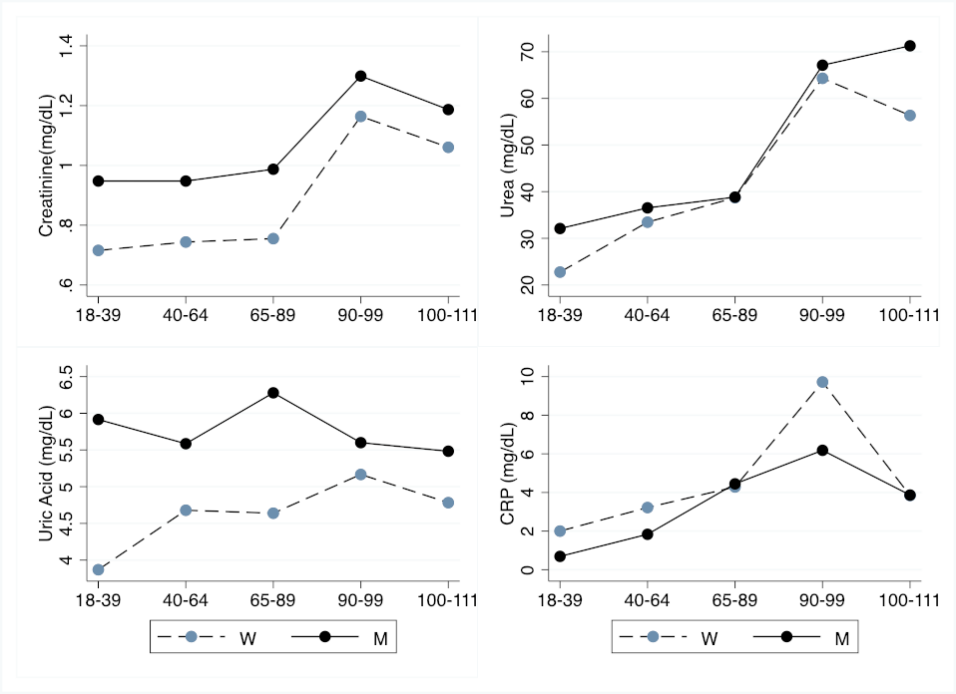
Hematochemical parameters: Catabolic and inflammatory parameters. The figure shows the trend of mean values by class of ages and gender related to catabolic and inflammatory parameters. Y-axis reports the mean values of the analysed biomarker, x-axis reported the age-class (for the acronyms see the table 10).

The tables 5 to 10 show hematochemical parameters, depicted graphically in the Figures 5 to 10, according to classes of age.

Concerning endocrine markers (Table 5; Fig. 5), FT3 is significantly lower in LLIs, whereas TSH and FT4 are not affected by age. No differences are observed for insulin and HOMA index as well as for glycaemia. About liver markers (Table 6; Fig. 6), ALT levels are significantly decreased both in female and male LLIs, so with a gender effect, whereas not significant differences are observed for AST and GGT. On the contrary, bilirubin and conjugated bilirubin show an age-related decrease in the whole population but significant from young to nonagenarian. Finally, albumin as well as total protein also show an age-related decrease.

Regarding iron markers (Table 7; Fig. 7), only iron levels show a significant decrease in LLIs, whereas there is a significant sex heterogeneity for ferritin since its values are lower in females.

Total cholesterol is not increased in LLIs when compared to other age groups, whereas triglycerides raise in nonagenarians only. In centenarians, HDL is not significantly different from values observed in all groups, whereas nonagenarians show lower levels. LDL levels of LLIs are not significantly different from those observed in young people. Adult and older people display, instead, levels significantly higher than those observed in young. No gender effect is registered (Table 8; Fig. 8).

As regards bone markers (Table 9; Fig. 9), in women there is a very significant age- and gender-related increase of osteocalcin. ALP shows a significant age-related increase, mostly in nonagenarian females, whereas calcium is significantly decreased in LLIs when compared to young adults and adults. Also, vitamin D levels are significantly decreased in LLIs when compared to young and adult groups. No effect of gender is observed. Magnesium is not significantly different between age groups and gender.

There is a significant increment of urea and creatinine in LLIs, whereas uric acid (UA) is unmodified, although there is a significant gender heterogeneity for UA since its values are lower in women. Lastly, in LLIs CRP is increased when compared to young and adult age groups but significance was attained only comparing the young population to nonagenarians (Table 10; Fig. 10).

**Table 9:** Hematochemical parameters: Bone parameters.

Variable (unit of measurement)	(a) Young Adults (18-39 y.o.) N=29; M=13; W=16	(b) Adults (40-64 y.o.) N=40; M=21; W=19	(c) Older adults (65-89 y.o.) N=54; M=28; W=26	(d) Nonagenarians (90-99 y.o.) N=27; M=9; W=18	(e) Centenarians (100-111 y.o.) N=23; M=6; W=17	Age	Interaction		Gender
							M	W		
Osteocalcin (ng/mL, 14-46)	23.85 (14.3-43.60) N=28, (25.98±8.56) M=13, (28.83±8.97) W=15, (23.50±7.63)	18.80 (8.62-32.50) N=40, (19.50±6.09) M=21, (17.59±4.95) W=19, (21.61±6.64)	20.20 (7.06-54.30) N=54, (21.88±8.88) M=28, (19.77±5.21) W=26, (24.14±11.30)	39.50 (12.40-217) N=27, (55.07±48.95) M=9, (39.09±26.31) W=18, (63.06±56.01)	39.10 (15.80-95.20) N=23, (44.60±24.14) M=6, (22.78±6.39) W=17, (52.29±23.39)	(a vs d) (b vs d,e) (c vs d)	ns	(a vs d,e) (b vs d,e) (c vs d,e)	<0.001
ALP (U/L, 40-129)	45.00 (25.00 -81.00) N=29, (48.34±12.98) M=13, (55.23±14.37) W=16, (42.75±8.64)	64.00 (32.00 -114.00) N=40, (65.90±17.54) M=21, (65.43±17.03) W=19, (66.42±18.55)	70.50 (35.00 -156.00) N=54, (72.24±24.23) M=28, (65.39±16.81) W=26, (79.61±28.82)	79.00 (57.00 -369.00) N=27, (102.59±73.92) M=9, (74.33±12.93) W=18, (116.72±87.43)	83.00 (53.00 -168.00) N=23, (88.30±24.07) M=6, (97.00±38.94) W=17, (85.24±16.87)	(a vs c,d,e) (b vs d)	ns	(a vs c,d,e) (c vs d)	ns
Calcium (mg/dL, 8.40-10.20)	9.54 (8.45-9.94) N=29, (9.48±0.30) M=13, (9.56±0.21) W=16, (9.42±0.36)	9.45 (8.71-10.40) N=40 (9.43±0.38) M=21 (9.45±0.36) W=19 (9.41±0.42)	9.26 (8.26-10.34) N=54, (9.26±0.41) M=28, (9.29±0.43) W=26, (9.25±0.41)	8.99 (8.02-10.11) N=27, (9.10±0.56) M=9, (8.94±0.25) W=18, (9.19±0.66)	9.04 (8.15-10.60) N=23, (9.00±0.59) M=6, (8.85±0.46) W=17, (9.06±0.64)	(a vs d,e) (b vs d,e)	ns	ns	ns
Magnesium (mg/dL, 1.60-2.60)	2.02 (1.74-2.24) N=29, (2.03±0.11) M=13, (2.07±0.13) W=16, (2.00±0.10)	2.07 (1.49-5.00) N=40, (2.14±0.49) M=21, (2.04±0.18) W=19, (2.12±0.16)	2.05 (1.66-2.42) N=54, (2.03±0.14) M=28, (2.03±0.15) W=26, (2.05±0.15)	2.05 (1.57-2.54) N=27, (2.08±0.23) M=9, (2.14±0.21) W=18, (2.06±0.25)	2.09 (1.49-2.57) N=23, (2.10±0.26) M=6, (1.96±0.30) W=17, (2.16±0.14)	ns	ns	ns	ns
Vitamin D (ng/mL, >30)	27.40(16.40-62.30) N=29, (29.70±9.89) M=13, (27.45±8.87) W=16, (31.53±10.56)	22.40(10.10-58.40) N=39, (24.08±9.36) M=21, (26.04±10.23) W=18, (21.78±7.90)	19.25(3.00-52.20) N=54, (20.91±10.06) M=28, (21.18±8.46) W=26, (20.62±11.71)	11.00(3.21-46.70) N=27, (14.67±12.01) M=9, (12.35±5.63) W=18, (15.82±14.19)	9.19(3.00-39.90) N=23, (11.82±9.38) M=6, (16.76±12.25) W=17, (10.08±7.85)	(a vs c,d,e) (b vs d,e)	ns	ns	ns

Abbreviations: y.o.=years old; N=total number of cases; M=men; W=women; SD=standard deviation; ALP=alkaline phosphatase. Data underlined are the median (min-max) of the total number of cases. Data between round brackets are mean values±SD. a, b, c, d, and e indicate, respectively, young adults, adults, older adults, nonagenarians, and centenarians. The table shows the Pairwise comparisons between the different groups, *i.e*., a, b, c, d, and e stratified for gender. p-value≤0.05 is considered significant; ns=not significant.

## DISCUSSION

Epidemiological data from various populations clearly suggest that centenarians represent an extraordinary and informative model for identifying the mechanisms responsible for healthy aging, although in most studies the genetic, demographic and phenotypic characteristics of longevity are discussed separately [7,22,28-31].

However, longevity is a complex trait due to the interactions of numerous genetic and environmental factors. Therefore, it is necessary to analyse the centenarians with a multidimensional approach, trying to consider different aspects at the same time, as performed in the present study [32].

We report anamnestic, anthropometric, molecular, haematological, oxidative, and hematochemical data of a homogeneous population of Sicilian centenarians and nonagenarians studied together with young adults, adults and older adults to have an adequate and matched number of controls. The information reported should be useful to understand the mechanisms that allow us to reach an advanced age in acceptable health conditions and to provide information on the reference ranges in older adults and LLIs, taking into account the gender.

Regarding anamnestic data, analysing LLIs diet, it is not closely adherent to the traditional MedDiet even if it is rich in bioactive foods such as fruit, vegetables, legumes, and EVOO and poor in red meat, so it is an anti-inflammatory diet [33-35]. It is noteworthy that control of inflammation is a property of the slow-aging diets able to slow the ageing process, delaying or preventing a range of chronic age-related diseases [36]. These diets are characteristics of people living in Blue Zones, regions of the world where a higher than usual number of people live much longer than average, becoming centenarians [37-40]. From the Sixties, the consumption of meat, fish, fats and sugars has significantly increased in Southern Italy, while the consumption of whole cereals and vegetables has decreased. It is, therefore, likely that during this nutritional transition there was a change in the diet of the LLIs, as in the rest of the Italians [41]. Thus, the LLIs of our cohort adhered to the MedDiet at a young age for food shortages rather than choice. Necessarily, the nutritional options were strictly seasonal and composed by local products only, so rich in nutraceuticals, and the amount of food was sufficient but never excessive, *i.e*., a kind of calorie restriction, well known for its pro-longevity effect [42]. These eating habits could affect ability to achieve extreme longevity through epigenetic modifications [33,41,43,44]. Concerning the other age groups, our results agree with a survey performed on 3,090 Sicilians that assessed low to moderate adherence to the traditional dietary patterns, particularly in the younger segment of Sicilian population [45].

**Table 10:** Hematochemical parameters: Catabolic and inflammatory parameters.

Variable (unit of measurement)	(a) Young Adults (18-39 y.o.) N=29; M=13; W=16	(b) Adults (40-64 y.o.) N=40; M=21; W=19	(c) Older adults (65-89 y.o.) N=54; M=28; W=26	(d) Nonagenarians (90-99 y.o.) N=27; M=9; W=18	(e) Centenarians (100-111 y.o.) N=23; M=6; W=17	Age	Interaction		Gender
							M	W		
Creatinine (mg/dL, 0.50-1.20)	0.82 (0.49-1.20) N=29, (0.82±0.16) M=13, (0.95±0.12) W=16, (0.72±0.10)	0.81 (0.55-1.37) N=40, (0.85±0.19) M=21, (0.95±0.15) W=19, (0.74±0.18)	0.83 (0.52-1.62) N=54, (0.88±0.24) M=28, (0.99±0.25) W=26, (0.76±0.15)	1.07 (0.61-2.73) N=27, (1.21±0.55) M=9, (1.30±0.44) W=18, (1.16±0.60)	1.06 (0.64-2.47) N=23, (1.09±0.41) M=6, (1.19±0.35) W=17, (1.06±0.43)	(a vs d,e) (b vs d,e) (c vs d,e)	ns	ns	ns
Urea (mg/dL, 16.6-48.5)	26.70 (16.20-46.00) N=29, (26.95±7.47) M=13, (32.10±6.10) W=16, (22.76±5.71)	33.85 (20.20-57.90) N=40 (35.08±9.77) M=21 (36.53±9.10) W=19 (33.47±10.47)	37.95 (19-60.20) N=54, (38.78±8.33) M=28, (38.83±9.62) W=26, (38.72±6.88)	47.80 (24.80-158.10) N=27, (65.24±36.16) M=9, (67.11±41.29) W=18, (64.31±34.56)	49.70 (30.80-142.30) N=23, (60.23±27.67) M=6, (71.27±40.77) W=17, (56.34±21.72)	(a vs d,e) (b vs d,e) (c vs d,e)	ns	ns	ns
Uric Acid (mg/dL, 2.4-7)	4.40 (2.80-7.50) N=29, (4.79±1.35) M=13, (5.92±1.12) W=16, (3.87±0.63)	5.10 (2.60-7.80) N=40, (5.16±1.26) M=21, (5.59±1.14) W=19, (4.68±1.23)	5.35 (2.20-8.30) N=54, (5.49±1.52) M=28, (6.28±1.31) W=26, (4.64±1. 26)	5.30 (2- 8.70) N=27, (5.31±1.74) M=9, (5.60±1.89) W=18, (5.17±1.71)	4.60 (3.10-8.60) N=23, (4.97±1.26) M=6, (5.48±1.81) W=17, (4.78±1.02)	ns	ns	ns	<0.001
CRP (mg/dL, <5)	0.62(0.12-9.51) N=28, (1.44±2.34) M=12, (0.69±0.58) W=16, (2.00±2.98)	1.70 (0.43-23.23) N=40, (2.49±3.57) M=21, (1.83±1.25) W=19, (3.22±4.98)	1.49 (0.23-63.86) N=54, (4.37±9.38) M=28, (4.45±12.02) W=26, (4.29±5.52)	3.07 (0.39-50.65) N=27, (8.54±12.00) M=9, (6.18±8.45) W=18, (9.72±13.50)	2.51 (0.37-18.34) N=23, (3.85±3.94) M=6, (3.87±1.70) W=17, (3.84±4.53)	(a vs d)	ns	ns	ns

Abbreviations: y.o.=years old; N=total number of cases; M=men; W=women; SD=standard deviation; CRP=C-reactive protein. Data underlined are the median (min-max) of the total number of cases. Data between round brackets are mean values±SD. a, b, c, d, and e indicate, respectively, young adults, adults, older adults, nonagenarians, and centenarians. The table shows the Pairwise comparisons between the different groups, *i.e*., a, b, c, d, and e stratified for gender. p-value≤0.05 is considered significant; ns=not significant.

About the other aspects of lifestyle of these Sicilian LLIs, Accardi et al., have reported that longevity mostly concerns people living in small villages, without pollution, likely because of different working conditions, different lifestyles, *i.e*., reduced smoking and alcohol abuse and an anti-inflammatory diet [46]. Longevity is observed particularly in small municipalities because individuals with greater access to social support and family networks have better health care and lower levels of mortality, particularly when adult daughters are present. Most of our LLIs live on the mountains in multi-storey homes, so throughout their life they are constantly in physical exercise. Indeed, it was demonstrated the positive association between increased levels of physical activity, a feature of the lifestyle of Blue Zone also, and improved health in older adults [40,47].

In relation to BMI, usually centenarians are thought to be underweight but, in our cohort, overall they show BMI not significantly different from values observed in young adults and lower than those observed in older adults. It is noteworthy that the values and the range are almost like those observed in a recent study of a large number of Portuguese centenarians [48]. These data are interesting considering that underweight and overweight conditions are unfavourable for longevity. This is the so-called obesity paradox that implies an inverse correlation between BMI and mortality, as demonstrated by several studies, although it could also be related to a low specificity of BMI for this segment of population [49,50]. This last hypothesis is reinforced by the analysis of body composition. It demonstrates lower PhA values in all classes of old individuals, both in men and in women, with significant differences between the genders. Using the PhA values, we should deduce that, on average, the centenarians under study are sarcopenic. From a theoretical point of view, the PhA data are not surprising because it is known that the higher are the PhA values, the better is the health condition [51]. PhA decreases as the body cell mass is lowered. Furthermore, it depends on extracellular water with inverse proportionality. A lower PhA appears to be compatible with cell death or a breakdown of the selective permeability of the cell membrane, in accordance with oedema. BIA specifically assesses hydration in any condition (clinical and otherwise) and is a suitable method for nutritional assessment. However, current models have been developed from analysis in healthy younger subjects. Therefore, as recently discussed, a note of caution should be added as it may not be fully suitable for the centenarian population likely suffering massive fluids and electrolysis changes [12,52].

Telomeres undergo shortening with each mitotic division and this process is modulated by inflammation and oxidative stress. The short telomeres therefore represent a marker of cumulative burden of inflammation and oxidative stress. Indeed, they are associated with a higher risk of all-cause mortality while individuals leading a healthy lifestyle have longer telomeres [53]. In our sample, LLIs women have RTL not significantly different from that of older women. Moreover, the RTLs of two semisuper- and super-centenarian sisters fit in the average plus/minus standard deviation of 60-69 Sicilian women [12].

The evolutionary conserved transcription factor FOXO3A plays important regulatory roles in insulin/insulin-like growth factor signalling. The activation of this pathway by a diet rich in proteins and refined sugars suppresses its transcription [54]. The FOXO3A rs2802292 is associated with longevity in different populations, likely due to an increased expression of FOXO3A involved in homeostatic responses [55]. However, in our cohorts we do not observe an association with longevity, likely for the small sample size although the MedDiet strictly followed at a young age by our LLIs might be responsible for the lack of association.

ApoE ε4 allele is a risk factor for the onset of Alzheimer’s and cardiovascular diseases. Hence, it has a deleterious effect on longevity. The ε3 allele is a neutral allele, whereas ε2 is the allele thought to promote longevity [56]. In our LLIs, we did not observe an association of ε2 with longevity, but we found the lowest percentage of ε4. These results are consistent with a recent analysis that shows that in South Italy there is a weaker protective effect of ε2 and no detrimental effect of ε4, suggesting that the MedDiet strictly followed in South Italy at a young age by the generations under study is responsible, as above, for that difference [56].

Heme synthesis declines with age and its deficiency should be responsible for the lower levels of red blood cells and hemoglobin in LLIs, although some underlying pathological conditions might be involved. The lower values of platelets in the LLIs, when compared to the young adults, are likely due to the slowing down of the haematopoietic system activity during aging [57-59].

No significant differences are observed in leukocyte counts. Neutrophils, traditionally considered as a component of acute inflammation, also increase in chronic age-related diseases, as atherosclerosis [60]. Lymphocytes are involved in immune responses and their age-related changes are represented by a different ratio of their subsets rather than an age-related decrease. N/L is an emerging inflammatory marker because it combines the predictive power of both decreased lymphocyte and increased neutrophil counts [26]. In people aged 55 years and older, this ratio is associated with mortality [61]. However, the not significant increase of N/L ratio observed in LLIs (and to a lesser extent in older adults) is related to the low-grade inflammatory status of old people called “inflammaging” [62,63].

Aging is associated with an increase in pro-oxidant factors and a decrease in antioxidant mechanisms. Oxidative stress plays an important role in determining and maintaining the typical low-grade inflammation, in turn contributing to oxidative stress. However, in several groups of centenarians, some indexes of oxidative stress have been demonstrated to be lower than in older subjects [64]. In centenarians, PON units are not significantly different from those observed in young people. PON is an enzyme associated with HDL, believed to protect against the oxidation (ox-) of LDL, so protecting from the risk of coronary artery disease [65]. It is noteworthy that the value range of ox-LDL in these centenarians is lower than that observed in young people [66]. MDA, the main product of the polyunsaturated fatty acids peroxidation, is not significantly different between the groups. The range values of blood glutathione in these centenarians have been shown to be included in the value range of young people [66]. It is seemingly puzzling that the total antioxidant capacity is lower in young people than in the other groups and that the highest values are observed in nonagenarians. As suggested, the eating habits of adults and older people, more adherent to MedDiet than young people could contribute to our observations [67].

Concerning hematochemical values, FT3 are significantly lower in LLIs. This result agrees with data that clinical or latent hypothyroidism is rising in the older population. A lower activity of thyroid hormone, and, thus, a decreased basal metabolic rate lowering oxidative metabolism might reduce DNA damage due to reactive oxygen species [68].

Regarding the decrease of ALT values, ALT levels decrease with age in both men and women independent on metabolic syndrome components, adiposity signalling biomarkers, and other commonly used liver function tests, although further studies are needed to understand the mechanisms responsible for its decline [69]. In our cohort, bilirubin shows a significant age-related decrease, except in centenarians. In contrast, the study of Boland et al., reveals that serum bilirubin levels modestly increase with age and that elevated bilirubin in older individuals is not associated with improved survival, as previously reported in middle-aged populations [70]. Albumin (and total proteins) levels are decreased in LLIs in agreement with results obtained in several centenarian studies. It has been used as a biochemical indicator of nutritional status. However, albumin levels are more a reflection of overall chronic or acute disease burden than of nutritional status, in particular their decrease is also linked to inflammatory status, as negative acute phase protein [71].

Iron deficiency anaemia is prevalent in older people, particularly after the age of 80. So, the significant serum level decrease in our LLIs is not surprising because chronic inflammation of older people makes the measurement of iron status difficult. Levels of circulating hepcidin, elevated in response to inflammation, are likely responsible for systemic iron depletion [66,63,72]. Other contributory factors could be the scarcity of iron content in the diet and the assumption of some medications, such as aspirin [73].

Our data on cholesterol, LDL and HDL, as well as triglycerides are substantially in line with the literature data. Indeed, studies indicate that total, LDL and HDL cholesterol levels of centenarians are either not different or lower than their older adult controls. Instead, triglycerides are like healthy older adult controls [71].

Regarding osteocalcin, an osteoblast-specific secreted protein expressed by mature osteoblasts, the observed increase is not surprising because it is used in clinical practice and in research as a marker of bone turnover [74]. The same meaning should have the age-related increase in ALP mostly due to female gender [75]. The low levels of calcium observed in LLIs are likely linked to low levels of vitamin D due to inadequate consumption or exposure to sunlight [76,77].

As expected, there is a significant increase of urea and creatinine values in LLIs, according to age-related progressive reduction of kidney functionality [59,78].

CRP is an acute phase reactant that responds rapidly to tissue injury, infection and inflammation. Not surprisingly, significantly higher concentrations of the protein are reported for LLIs as compared with their controls (although the increase was not significant in centenarians), in the present study, as well as in several other reports [71].

The decrease of albumin levels, the increase of CRP levels as well as not significant increase of N/L ratio in LLIs are witnesses of the chronic inflammatory state of the LLIs. In addition, our LLI cohort has been shown to present the kynurenine/tryptophan (Kyn/Trp) ratio, a valuable marker for the rate of inflammaging, higher than in all other age-groups [66,79].

Inflammaging is a process observed in any older subjects. However, centenarians seem to be equipped with gene variants that optimize the balance between pro- and anti-inflammatory molecules, minimizing the detrimental effect of inflammaging [80]. The increased plasma levels of pro-inflammatory molecules should be counter-balanced by a higher level of anti-inflammatory molecules. LLIs belonging to this cohort displayed an increased enzymatic activity of the extracellular proteinase matrix metalloproteinase 2 known to regulate intercellular communication, including inflammation [81]. The value range of microRNA miR-223-5p, involved in the control of inflammation, in the centenarians under study is higher than that observed in young and adult people [66,82]. These two observations suggest a possible epigenetic modulation, with anti-inflammatory effects, that confer protection against tissue damage [83].

An important evidence of the present study is that there are differences related to both age and gender in several biomarkers. In fact, despite their biomedical relevance, gender differences seem to be still poorly considered and inadequately investigated in medical studies including aging [84,85].

Moreover, it is to note that often we observed comparable parameters between young and centenarians rather than nonagenarians and centenarians, hypothesizing a sort of slowdown in the decay of systemic deterioration.

The small sample size is a limitation of the present study, mostly regarding the molecular tests, although performed in a very homogeneous population. Thus, due to the small number of individuals studied, the data concerning the various biomarker values should be utilized and validated in further studies.

Much remains to be learned about the prognostic significance of these biomarkers and their possible treatment. Particularly, if it makes sense to treat the pro-inflammatory state of LLIs. Would such treatment lead to improvements in physical, cognitive and/or functional state of LLIs? How might knowledge of the RTL be used to decrease morbidity and delay mortality in late life? Addressing these and other questions regarding the interpretation of biomarkers in LLIs is increasingly important as many people cross the 90 years. The identification of the factors that predispose to healthy life in a relatively good health status, *i.e*., a longevity signature, is of enormous interest for translational medicine in an aging world.

A long life in a healthy, vigorous, youthful body has always been one of humanity’s greatest dreams. Slow the ageing process means not only to delay or avoid the onset of age-related disease but also to extend the year of life free from the need of care and being independent, with a direct effect on the cost of healthcare. The possibility to target aging directly, instead of treating age-related pathologies could be of great relevance to improve the quality of life worldwide and to guarantee a healthy longevity in the light of positive biology [4,86].

## Supplementary Materials

The Supplemenantry data can be found online at: www.aginganddisease.org/EN/10.14336/AD.2021.0226.


