# Supplementary material for: Age and Gender-related Variations of Molecular and Phenotypic Parameters in A Cohort of Sicilian Population: from Young to Centenarians
Source: Aging Dis. 2021 Oct 1;12(7):1773–93. doi: 10.14336/AD.2021.0226 (PMC8460304; doi:10.14336/AD.2021.0226)
Supplement: Supplementary file 1 [file AD-12-7-1773-s.pdf]

SUPPLEMENTARY DATA

Age and Gender-related Variations of Molecular and Phenotypic Parameters in A Cohort of Sicilian Population: from Young to Centenarians

Anna Aiello[1,#], Giulia Accardi[1,#], Stefano Aprile[1,2], Rosalia Caldarella[3], Ciriaco Carru[4], Marcello Ciaccio[3,5], Immaculata De Vivo[6], Caterina Maria Gambino[5], Mattia Emanuela Ligotti[1], Sonya Vasto[7], Angelo Zinellu[4], Calogero Caruso[1*], Filippa Bono[8*], Giuseppina Candore[1*]

© 2021. Aiello A et al. Published online at http://www.aginganddisease.org/EN/10.14336/AD.2021.0226

Project: DESIGN

Questionnaire

Sample code:

Name of interviewer:

© 2021. Aiello A et al. Published online at http://www.aginganddisease.org/EN/10.14336/AD.2021.0226

SUPPLEMENTARY DATA

Anagraphic data

Date of visit ______

Surname ______

Name ______

Sex [M] [F]

Place of birth ______

Date of birth ______

(n° of ID) ______

Home address ______

Second address ______

Phone ______

Are your parents alive? YES NO Mother Father

Parents age (if alive) ______

NOTE specify the date and the cause of death of parents, uncles, brothers and sisters

NOTE specify how many people are in your immediate family, if they are alive or not and which was the cause of death (eventually and for old and centenarians only).

Name, surname, phone and address of your family doctor:

[se 4-9]

Do you have a live-in caretaker? (only for old and centenarians)

1. Spouse
2. Brothers/sisters
3. Sons/Daughters
4. Other parents
5. Friends
6. Nursing home
7. Caretaker

Do you live in a:

1. Home
2. Nursing home
3. Other

Tax status:

1. Single
2. Married
3. Widow/er
4. Divorced

Education

Never go to school (self-educated)
Primary school (not finished)
Primary school
Middle school
High school
Bachelor/Master's degree
PhD
Speciality (post-lauream)
Illiterate

Job (actual A and past P)

Manager
Employed (sedentary. Es. office work)
Employed (active. Es. postoffice)
Farmer, manual labor
Self- employed
Housewife
Other (specify)………

Personal income (pre e post retirement)

PRE	POST

© 2021. Aiello A et al. Published online at http://www.aginganddisease.org/EN/10.14336/AD.2021.0226

Very low		
Low		
Good		
High		
Very high		

Place of interview ________________________

© 2021. Aiello A et al. Published online at http://www.aginganddisease.org/EN/10.14336/AD.2021.0226

SUPPLEMENTARY DATA

Cognitive and functional state (Only for LLIs)

Mini mental state examination (MMSE)

(1=YES; 0=NO)

**Mini-Mental State Examination**	
What is the year? (0-1)	
What is the season? (0-1)	
What is the month? (0-1)	
What date is today? (0-1)	
What is the day (of the week) is? (0-1)	
Which nation is? (0-1)	
What region is? (0-1)	
What city is? (0-1)	
Where are we? (0-1)	
What floor is? (0-1)	
Please, repeat: “Home, bread, cat” (0-3)	
I would like you to count backward from 100 by sevens (93-86-79-72-65). Alternative: “Spell WORLD (D-L-R-O-W) (0-5)	
Earlier I told you the names of three things. Can you tell me what those were (0-3)	
Show the patient two simple objects, such as a wristwatch and a pencil, and ask the patient to name them (0-2)	
Repeat the phrase: “Tiger against tiger” (0-1)	
“Take the paper in your right hand, fold it in half, and put it on the floor.” (The examiner gives the patient a piece of blank paper.) (0-3)	
Please read this and do what it says.” (Written instruction is “Close your eyes.” (0-1)	
**“Make up and write a sentence about anything.” (This sentence must contain a noun and a verb.) (0-1)** ______ ______	

**Please copy this picture (0-1)** 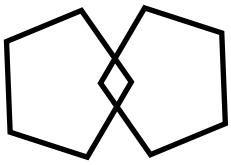		
**Tot score MMSE**		
**Not applicable**		

© 2021. Aiello A et al. Published online at http://www.aginganddisease.org/EN/10.14336/AD.2021.0226

Activity of daily living (ADL) (Only for LLIs)

	Yes	No
Are you able to feed yourself?		
Are able to leave your bed by yourself?		
Are you able to dress by yourself?		
Are you able to use the toilette by yourself?		
Are you able to take a shower or a bath by yourself?		
Are you able to control over bodily functions? (if no, please indicate since when you use catheter or adult diaper)		

Instrumental activity of daily living (IADL) (Only for LLIs)

	Yes	No
Do you usually cook your meals by yourself?		
Do you usually do regular housekeeping?		
Do you usually use means of transportation?		
Do you usually go shopping?		
Do you usually do laundry?		
Do you usually use the phone?		
Do you usually take medicine alone?		
Do you usually manage your financial matters?		

© 2021. Aiello A et al. Published online at http://www.aginganddisease.org/EN/10.14336/AD.2021.0226

SUPPLEMENTARY DATA

Nutrition

Have you lost your appetite in the last year?
YES ☐ No ☐
Have you eaten less in the last three months?
1. High reduction of food consumption ☐
2. Moderate reduction of food consumption ☐
3. No reduction of food consumption ☐

Are you eating a well-balanced diet?
1. High malnutrition ☐
2. Moderate malnutrition or do not know ☐
3. No nutritional problem ☐

Have you lost weight in the last 2/5 years?
Yes ☐ No ☐
If yes, how many kilos?__________________
4. Do you have teeth? Yes No

Smoke and alcohol

Never smoked ☐
Prevoius smoker (n° of cigarettes) ☐
How many years did you regularly smoke? ☐
Smoker (n° of cigarettes) ☐
Have you ever quit smoking? ☐
If yes, for how long? ☐

Do you drink alcohol (es. hard liquor or wine or beer)? ☐
If yes, how many glasses a week? ☐

What kind of alcoholic beverages do you drink?

Wine? (Please, indicate if red:R or white:W (B in italian) ☐
Beer? ☐
Hard liquor? ☐

Sleep

At what time do you go to bed? ______________
How many hours do you sleep? __________
Do you get up during the night? Yes No

Are you satisfy by your sleep? Yes No

Do you do nap during the day? Yes No

Yes No

Do you take some medicines to sleep? ☐ ☐

Do you remember your dreams? Yes No

Do you have trouble falling asleep? Yes No
Do you snore? Yes No

Have you ever had insomnia? Yes No
If yes, when (at what age)? ______________

Religion

Are you religious?
1. Yes, I go to the church or I pray everyday ☐
2. Yes, I watch church services on TV on Sunday ☐
3. I'm believer but no practicing ☐
4. I'm not believer ☐

Health and morbidity

How you define your health status:
1. Excellent ☐
2. Very good ☐
3. Good ☐
4. Bad ☐
5. Very bad ☐

Have you fallen down in the last year? YES NO
If yes, how many times ___________

Have you were in the hospital in the last year?
YES NO
If yes, how many times ___________

Have you got fractures in the last year? YES NO
If yes, in which part of your body
____

Have you bedsores or skin ulcer? YES NO

Diseases

Cardiovascular (past/present) Yes No

© 2021. Aiello A et al. Published online at http://www.aginganddisease.org/EN/10.14336/AD.2021.0226

SUPPLEMENTARY DATA

	Yes	No
1. Hypertension		
2. Ischemic heart disease		
3. Supra-aortic trunks ateromasia		
4. Pulmunary embolism		
5. Atrial fibrillation		
6. Deep vein thrombosis		
7. Orthostatic hypotension		
8. Peripheral arterial disease		
9. Claudicatio		
10. Heart failure		

Neurological (past/present)

	Yes	No
1. Stroke		
2. Ictus		
3. Hemiparesis/Hemiplegia		
4. Tetraplegia		
5. Parkinson's disease		
6. Epilepsy		

Gastrointestinal (past/present)

	Yes	No
1. Gastritis		
2. Ulcer		
3. Dyspepsia		
4. Constipation		
5. Diarrhoea		
6. IBD		
7. Diverticulosis/diverticulitis		
8. Colon poliposis		

Endocrine (past/present)

	Yes	No
1. Type II Diabetes		
2. Hypothyroidism		
3. Hyperthyroidism		

Musculoskeletal (past/present)

	Yes	No
1. Artrosis		
2. Osteoporosis		

Pulmunar (past/present)

	Yes	No
1. Asthma		
2. Enphysema		
3. Chronic obstructive pulmunary disease		

Sense (past/present)

	Yes	No
1. Glaucoma		
2. Deafness		
3. Macular degeneration		
4. Cataract		

Genitourinary (past/present)

	Yes	No
1. Urinar tract infection		
2. Dialysis		
3. Prostatic Hypertrofy		
4. Kidney failure		

Infectious (past/present)

	Yes	No
1. Fever		
2. Infection		

Cancer (past/present)

	Yes	No
1. Non metastatic tumor		
2. Metastatic tumor		

© 2021. Aiello A et al. Published online at http://www.aginganddisease.org/EN/10.14336/AD.2021.0226

SUPPLEMENTARY DATA

Medicines

Please, indicate the following information:
Commercial name
Dose
Per day (D)/per week (W)
Over the counter (OTC)
Occasional consumption (OC)

Name	Dose	D/W	OTC	OC

Verify the consume of laxatives.

© 2021. Aiello A et al. Published online at http://www.aginganddisease.org/EN/10.14336/AD.2021.0226

GERIATRIC DEPRESSION SCALE (Only for LLIs)

Choose the best answer for how you have felt over the past week:

1. Are you basically satisfied with your life? YES / NO
2. Have you dropped many of your activities and interests? YES / NO
3. Do you feel that your life is empty? YES / NO
4. Do you often get bored? YES / NO
5. Are you in good spirits most of the time? YES / NO
6. Are you afraid that something bad is going to happen to you? YES / NO
7. Do you feel happy most of the time? YES / NO
8. Do you often feel helpless? YES / NO
9. Do you prefer to stay at home, rather than going out and doing new things? YES / NO
10. Do you feel you have more problems with memory than most people? YES / NO
11. Do you think it is wonderful to be alive now? YES / NO
12. Do you feel pretty worthless the way you are now? YES / NO
13. Do you feel full of energy? YES / NO
14. Do you feel that your situation is hopeless? YES / NO
15. Do you think that most people are better off than you are? YES / NO

One point for "No" to questions 1, 5, 7, 11, 13

One point for "Yes" to the other questions

A score > 5 points is suggestive of depression.

A score ≥ 10 points is almost always indicative of depression.

© 2021. Aiello A et al. Published online at http://www.aginganddisease.org/EN/10.14336/AD.2021.0226

SUPPLEMENTARY DATA

-For men:
Do you remember your weight at 30 years?
(if yes, please indicate it)
____

-For women:
Age at menarche: ________
Age at menopause: ________
N° of pregnancies/abortion: _______
Last pregnancy (age): ________
Weight pre-pregnancy?
____

Physical function (code 7-9)

How far is your home to your office?
Less than 5 Km ☐
Between 5 Km and 15 Km ☐
More than 15 Km ☐
Other __________________

How do you go at work?
Foot ☐
Bicycle ☐
Motorbike or scooter ☐
Car ☐
Public transportation (bus, metro, train) ☐
Other (specify)………………………………………………….)

If you choose public transportation (bus, train, metro) to go to work, how far is the stop from your house?
Less 100 metres ☐
From 100 to 500 metres ☐
More than 500 metres ☐

Do you practice physical activity regularly?
Yes ☐
No ☐

If yes, what kind of activity do you practice? (it is possible more than one answer)
Running ☐
Walking ☐
Soccer ☐
Fitness ☐
Swimming ☐
Bicycle ☐
Other (specify)

If you practice physical activity, how many days a week?
Everyday ☐
Some days (please indicate ………) ☐
Only the week-end ☐

If you practice physical activity, how much time?
Less than 1 hour ☐
1 hour ☐
2 hours ☐
more than 2 hours ☐

Considering your daily working activities, how much time do you walk in a day?
Less than 30 minutes ☐
From 30 to 60 minutes ☐
More than 60 minutes ☐

Balance

0. It can't stay stand with your feet together for 10 seconds	
1. It can stand with your feet together for 10 seconds but not hips width apart	
2. It can stand with your hips width apart for 10 seconds but not with feet together	
3. It can stand with feet together for more than 2 second but less than 10 seconds	
4. It can stay in tandem for more than 10	
**Score**	

© 2021. Aiello A et al. Published online at http://www.aginganddisease.org/EN/10.14336/AD.2021.0226

© 2021. Aiello A et al. Published online at http://www.aginganddisease.org/EN/10.14336/AD.2021.0226

FOOD FREQUENCY QUESTIONNAIRE

Food Habits

(Don't fill out the meals that usually the subject doesn't consume)

A. Which meals do you usually consume in one day? And where?

	Home	Cafeteria/Office	Fast-food Chains	Pizzeria/Restaurant	Other
Breakfast					
Morning snack					
Lunch					
Afternoon snack					
Dinner					

B. If you eat for breakfast, what do you usually eat? (more answers allowed)

Coffee ☐ Brioche ☐ Milk ☐ Cookies ☐ Cappuccino ☐

Cereal ☐ Tea ☐ Italian dry bread ☐ Fruit juice ☐

Bread ☐ Yogurt ☐ Other ______________________________

C. If you eat for lunch, what do you usually eat? (more answers allowed)

☐ First serving ☐ Second serving ☐ A side of vegetables ☐ Bread ☐ Fruits ☐ Dessert ☐ Coffee

Other______________________________

D. If you eat for dinner, what do you usually eat? (more answers allowed)

☐ First serving ☐ Second serving ☐ A side of vegetables ☐ Bread ☐ Fruits ☐ Dessert ☐ Coffee

Other______________________________

E. During the morning snack, what do you usually eat? (more answers allowed)

☐ The ☐ Coffee ☐ Fruit juice ☐ Fresh Fruits ☐ Dry Fruits ☐ Cookies ☐ Yogurt

Other______________________________

F. During the afternoon snack, what do you usually eat? (more answers allowed)

☐ ☐ ☐ ☐ ☐ ☐ ☐

SUPPLEMENTARY DATA

The Coffee Fruit juice Fresh Fruits Dry Fruits Cookies Yogurt

Other____

I. How much water do you drink in one day (Please refer to the last year)?

☐ ≤ 0,5 L ☐ 0,5 L < x < 1 L ☐ 1 L < x < 1,5 L ☐ 1,5 L < x < 2 L ☐ > 2 L

L. How frequently do you consume sweetened beverages (fruit juices, tea, carbonated beverages)?

☐ A glass a day ☐ More than 3 glasses a day ☐ 2-3 times a week

☐ Once a month ☐ Rarely ☐ Never

M. How frequently do you consume alcoholic beverages?

☐ A glass a day ☐ More than 3 glasses a day ☐ 2-3 times a week

☐ Once a month ☐ Rarely ☐ Never

N. Do you add sugar to beverages? Yes No If yes, how? ___________________________

O. How frequently do you use this kind of cooking methods?

	**Two times in one day (lunch and dinner)**	**One time in a day (lunch or dinner)**	**Two-Three times in one week**	**One time in a week**	**One time in a month**	**Less than one time in a month**	**Never**
Boiled							
Steam							
Stew (cook on low heat)							
Oven							
Stir-fry							
Fried							
Roasted (pan or grilled)							
Grilled							

P. Do you follow a diet? (Es. vegeterian, etc.) ☐ Yes ☐ No If Yes, which one? __________

Q. Are you celiac? ☐ Yes ☐ No

R. Do you have food allergies? ☐ Yes ☐ No If Yes, which one? __________

S. Do you use dietary supplements? Yes No

SUPPLEMENTARY DATA

T. If Yes, how often? Occasionally Regularly

U. If Yes, which one? (More answers allowed) Vitamins | Minerals | Probiotics | Proteins/aminoacids | Dietary fibers | Antioxidants

Historic frequency of consumption for food groups (only for LLIs)

During the childhood (from the birth to 15 years old) you usually ate

	**Two times in one day (lunch and dinner)**	**One time in a day (lunch or dinner)**	**Two-Three times in one week**	**One time in a week**	**One time in a month**	**Less than one time in a month**	**Never**
Milk, cheese, yogurt							
Legumes (please specify)							
Pasta or Bread (please specify which one)							
Meat (please specify which one)							
Eggs							
Seafood							
Fruits or vegetables (please specify which one)							
Dessert							

Frequency of consumption for food groups IN THE LAST MONTH

	**Two times in one day (lunch and dinner)**	**One time in a day (lunch or dinner)**	**Two-Three times in one week**	**One time in a week**	**One time in a month**	**Less than one time in a month**	**Never**
**Cereals and Derivatives and Tubers**							
Pasta							
Rice							
Bread							
Pizza							
Spelt							
Barley							
Potatos							
Other (specify)							

SUPPLEMENTARY DATA

	**Cereal-Based Foods**						
Breakfast cereals							
Italian dry bread							
Cracker							
Other (specify)							

	**Two times in one day (lunch and dinner)**	**One time in a day (lunch or dinner)**	**Two-Three times in one week**	**One time in a week**	**One time in a month**	**Less than one time in a month**	**Never**
**White Meat**							
Chicken							
Turkey							
Rabbit							
Lamb							
**Red Meat**							
Veal							
Beef							
Pork							
**Processed Meat**							
Ham, etc.							
Salami, wurstel							
Canned meat							
**Eggs**							
Eggs from chicken							
Other							
**Seafood**							
Bluefish (gilthead bream, mackerel, anchovy, etc)*							
Crustaceans							
Shellfish							
Canned seafood (tuna/ mackerel, smoked salmon)							
*F=Fresh; S=Frozen **Legumes**							
Dry Legumes							
Fresh Legumes							
Canned Legumes							
Frozen Legumes							

SUPPLEMENTARY DATA

**Dressing**							
Extra Virgin Olive oil							
Lemon							
Vinegar							
Salt							
Spices							
Aromatic Herbs							
Animal Fats (butter, lard, cream)							
Margarina							
Altro							

	**One portion in a day (lunch or dinner)**	**Two portions in one day (lunch and dinner)**	**2-3 portions in one week**	**1 portion in one week**	**One time in a month**	**Less than one time in a month**	**Never**
**Milk and milk derivatives**							
Cow’s milk							
Vegetable Milk (specify the kind)							
Yogurt from milk							
Yogurt from soy milk							
Others yogurt							
Fresh Cheese							
Aged cheese (Parmesan, pecorino, ecc.)							
**Sweeteners**							
Sugar							
Honey							
Sweetener (specify)							

**Desserts**								
Cookies								
Bakery products								
Gelato								
**Fruits**								
Seasonal Fresh Fruits								
Fresh fruits								
Exotic Fresh fruits								

SUPPLEMENTARY DATA

Dry fruits								
	**Two times in one day (lunch and dinner)**	**One time in a day (lunch or dinner)**	**Two-Three times in one week**	**One time in a week**	**One time in a month**	**Rarely**	**Never**	**No in this season**
**Vegetables**								
Tomato								
Pepper								
Beet								
Turnip								
Onions								
Cauliflower								
Fennel								
Mushroom								
Broccoli								
Zucchini								
Artichoke								
Pickle								
Eggplant								
Specify se di stagione o no								
Cabbage								
Radicchio								
Arugola								
Lattuce								
Spinach								
